# Knowledge and Attitudes Regarding Respiratory Syncytial Virus (RSV) Prevention: A Systematic Review

**DOI:** 10.3390/vaccines13020159

**Published:** 2025-02-06

**Authors:** Teresa Gavaruzzi, Andrea Ceccarelli, Camilla Nanni, Carloalberto Vignali, Valentina Colonnello, Marta Caserotti, Matteo Riccò, Davide Gori

**Affiliations:** 1 Department of Medical and Surgical Sciences, University of Bologna, 40138 Bologna, Italy; valentina.colonnello@unibo.it; 2 Department of Biomedical and Neuromotor Sciences, University of Bologna, 40126 Bologna, Italy; andrea.ceccarelli4@studio.unibo.it (A.C.); carloalberto.vignali@studio.unibo.it (C.V.); davide.gori4@unibo.it (D.G.); 3 Romagna Local Health Authority, 47121 Forlì, Italy; nanni.camilla98@gmail.com; 4 Department of Developmental Psychology and Socialization, University of Padova, 35131 Padova, Italy; marta.caserotti@unipd.it; 5 AUSL–IRCCS di Reggio Emilia, Servizio di Prevenzione e Sicurezza Negli Ambienti di Lavoro (SPSAL), Local Health Unit of Reggio Emilia, 42123 Reggio Emilia, Italy; matteo.ricco@ausl.re.it

**Keywords:** respiratory syncytial virus (RSV), prevention, attitudes, knowledge, intention, acceptance, vaccination, immunization, monoclonal antibody, systematic review

## Abstract

**Background**: New strategies for respiratory syncytial virus (RSV) prevention are available and are in development, but their acceptance is crucial to their effectiveness. Objectives: This systematic review aims to summarize current quantitative and qualitative evidence regarding knowledge and attitudes relating to RSV prevention. **Methods**: Six databases (PubMed, Scopus, APA PsycArticles; APA PsycInfo; CINAHL Complete; Psychology and Behavioral Sciences Collection) and two preprint repositories (medRxiv and Preprints) were searched up until 23 December 2024 (PROSPERO: CRD42024602351). **Results**: Sixty-one articles were included, focusing on vaccination for the elderly and adults at risk (n = 10) or pregnant people (n = 24, of which 8 also examined preferences for maternal vs. infant immunization) and infant immunization (n = 27, of which 16 focused on palivizumab, with 6 focusing on adherence to its monthly administration). Eighteen articles assessed attitudes in healthcare professionals. Overall, findings showed limited knowledge and awareness of RSV but generally positive attitudes towards prevention strategies and moderate to high intentions and uptake rates. Protection against the disease and perceived severity promoted acceptance, whereas concerns about side effects hindered it. Maternal vaccination was more acceptable than infant immunization. **Conclusions**: Attitudes towards RSV prevention options were generally favorable. Should more options become available, preferences may depend on which options are available, their characteristics, and how they are framed and presented. These insights highlight the importance of education on RSV grounded in decision-making literature, while recognizing the likely favorable reception of preventive measures across target age-populations.

## 1. Introduction

The human respiratory syncytial virus (RSV) is a respiratory virus from the genus *Orthopneumovirus* within the *Pneumoviridae* family [1,2,3,4]. RSV is responsible for approximately 33 million cases of acute lower respiratory tract infections (such as bronchiolitis and pneumonia) globally and is recognized as a common cause of morbidity, particularly among young children. Nearly all children contract RSV by age two, most experiencing only mild respiratory symptoms, though the virus follows a clear seasonal pattern [5,6]. In infants, while approximately 90% of cases are managed in outpatient settings, RSV is a significant driver of hospital admissions, even in otherwise healthy infants [7,8]. Additionally, RSV affects more than just pediatric populations; in fact, it also poses a substantial health burden for adults, especially immunocompromised individuals and older adults, with a marked impact on those in institutional care [9,10,11]. Consequently, RSV has a considerable global impact, both in direct healthcare costs and through indirect economic effects [12,13,14].

Several technological advancements have been developed to prevent RSV infections, particularly targeting the most vulnerable populations (i.e., young children and the elderly). Passive immunization with monoclonal antibodies including newer long-acting antibodies has become a key strategy for protecting infants, especially those at high risk. These antibodies provide temporary immunity by neutralizing the virus and preventing its spread in the respiratory tract [15,16,17]. In addition, subunit protein-based vaccines and innovative mRNA technology-based vaccines designed to elicit immune responses in both pediatric and elderly populations are undergoing advanced clinical trials, focusing on the RSV fusion protein (the key pathogenetic factor) to generate a robust immune response [18,19,20,21,22,23,24,25].

The approval of RSV vaccines and long-acting antibodies by major regulatory bodies, such as the U.S. Food and Drug Administration (FDA) and the European Medicines Agency (EMA), has marked a significant advancement in RSV prevention. In 2023, the FDA approved the use of RSV vaccines for older adults [26], while both maternal vaccines and long-acting monoclonal antibodies have been approved for newborns and children [27], offering protection during the peak RSV season. The EMA has similarly endorsed these innovations, paving the way for broader adoption across Europe. Several countries, including the United States and members of the European Union, have either introduced or are preparing to roll out these preventive measures, prioritizing high-risk populations such as infants, pregnant women, and the elderly. The increasing global focus on RSV prevention through these approvals reflects a commitment to reducing the virus’s impact, particularly among those most vulnerable to severe outcomes. On the other hand, it is well known that the availability of an approved vaccine does not guarantee its uptake by the target population: while the causes are likely multifactorial, vaccine hesitancy is a contributing factor. The most frequently studied factors that facilitate or inhibit preventive therapies utilization are structural barriers such as cost, availability, and accessibility. These barriers are already known and are considered by program implementers. In addition to these factors, the vaccine community must also consider vaccine hesitancy, an increasingly common phenomenon that impacts both immunization rates and the ability of a vaccine to reach its full potential [28]. Understanding people’s beliefs and opinions about RSV disease prophylaxis could be crucial for addressing the phenomenon of vaccination hesitancy and supporting health authorities to design tailored health policies and/or public health interventions regarding other RSV prophylactic therapies as highlighted in a 2022 systematic review that aimed to identify and understand global predictive factors for COVID-19 vaccination uptake [29].

This paper aims to summarize current evidence concerning knowledge and attitudes regarding RSV prevention, including intention to get vaccinated or uptake (when vaccines were already offered) and healthcare professionals’ recommendations. It examines these attitudes in terms of beliefs, perceptions, opinions, views, acceptance, interest, and preference.

## 2. Materials and Methods

This systematic review followed Preferred Reporting Items for Systematic Reviews and Meta-Analyses (PRISMA) guidelines and was registered in PROSPERO (CRD42024602351).

### 2.1. Search Strategy

**Figure 1:**
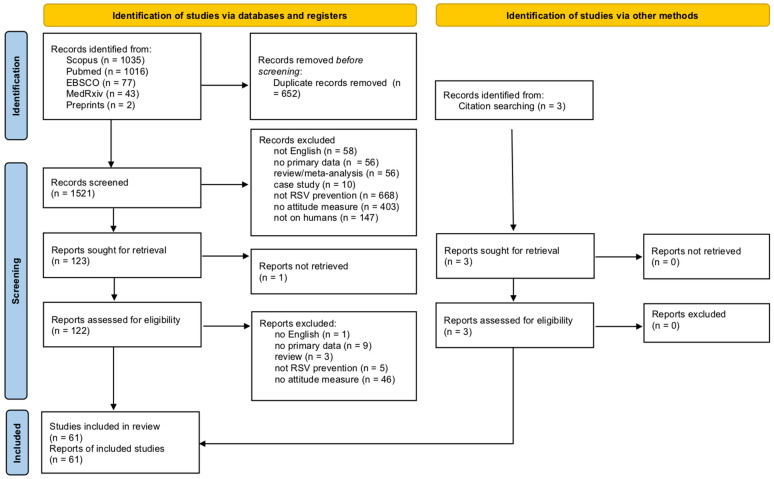
Flow diagram of the selection of articles. Preferred Reporting Items for Systematic Reviews and Meta-Analyses (PRISMA) diagram.

Articles were searched for directly in two databases (PubMed and Scopus), two preprint repositories (medRxiv and Preprints), and in four additional databases through the EBSCO platform (APA PsycArticles; APA PsycInfo; CINAHL Complete; Psychology and Behavioral Sciences Collection) up until 23 December 2024 (Figure 1) (see Supplementary Material for full search strategy). Additionally, hand searches were conducted by reviewing articles that cited the included articles and related articles. No further studies were identified through other methods. Three authors (CN, CV, TG) independently screened all articles, with divergences resolved through discussion with a third author (AC).

### 2.2. Inclusion and Exclusion Criteria

Articles were included if they reported primary data (quantitative, qualitative or both) on knowledge and any form of attitudes (including uptake or intention to get vaccinated or intention to recommend vaccination, beliefs, perceptions, opinions, views, acceptance, interest, and preference) regarding RSV prevention. Articles were eligible if they were published or a preprint, the full text was available, and they were written in English. Articles were excluded if they were not in English, were not full articles, did not report primary data, were not about RSV prevention, and did not assess attitudes towards RSV prevention.

### 2.3. Coding of Articles

Four authors (CN, CV, TG, VC) coded the articles independently, with divergences resolved through discussion with a third author (AC). From each article, the following characteristics were extracted: the aim of the study, country/countries where the data were collected, the period of data collection, the type of sample recruited (e.g., parents or healthcare professionals), sample size, the type of prevention discussed (RSV vaccines for elderly people and adults at risk or for pregnant women, or antibodies for infants), whether vaccines or antibodies were already offered or intended for future use, whether the article assessed attitudes towards RSV only or also other illnesses (e.g., COVID-19, influenza, etc.), the type of methodology used (quantitative, qualitative, or both) and study design (when applicable), the main results, and the strengths and limitations.

Based on the type of prevention discussed, articles were classified into one of three groups: (a) knowledge and attitudes regarding RSV vaccines for elderly people and adults at risk; (b) knowledge and attitudes regarding maternal RSV vaccines; and (c) knowledge and attitudes regarding RSV immunization for infants. For articles about maternal vaccination, it was noted whether attitudes towards immunization for infants were also assessed. For articles about infant immunization, it was noted whether they concerned palivizumab or not.

## 3. Results

The search process is detailed in Figure 1. Briefly, a total of 2173 articles were retrieved. After manually removing 652 duplicates, 1521 articles were screened for inclusion. Of these, 58 articles met the inclusion criteria. Three additional articles were identified using a hand search, for a total of 61 articles included in this review [30,31,32,33,34,35,36,37,38,39,40,41,42,43,44,45,46,47,48,49,50,51,52,53,54,55,56,57,58,59,60,61,62,63,64,65,66,67,68,69,70,71,72,73,74,75,76,77,78,79,80,81,82,83,84,85,86,87,88,89,90] (Table S1).

**Figure 2:**
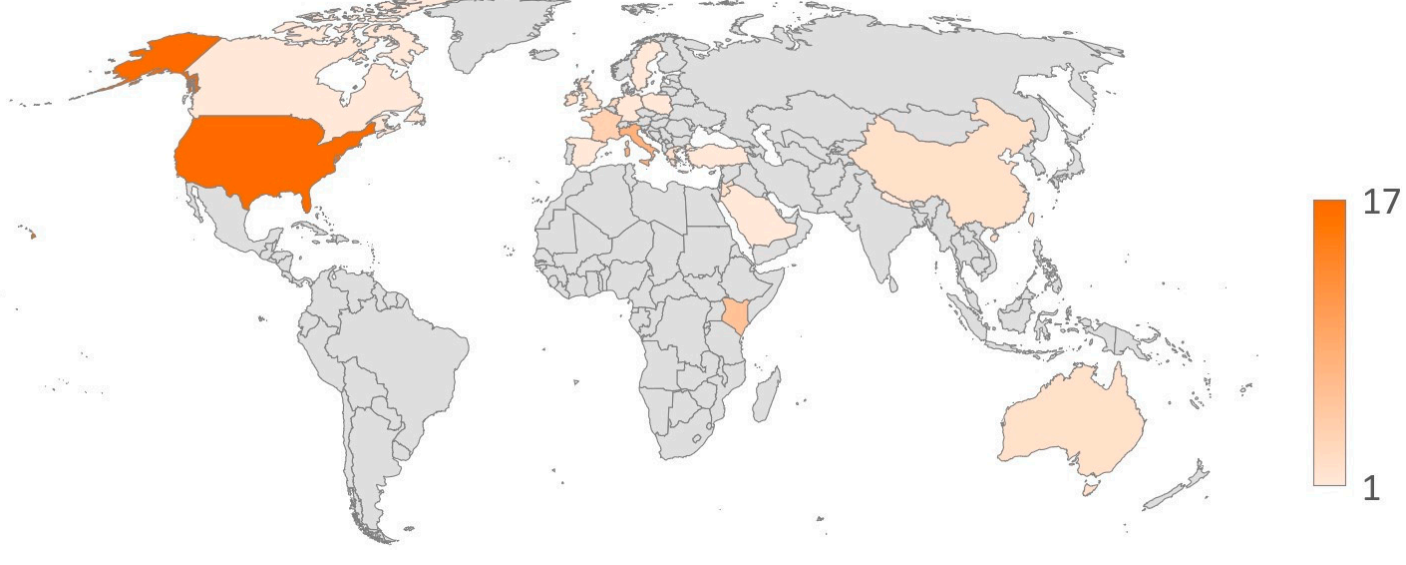
Distribution of countries where data were collected for articles included in this review. This figure represents the number of articles (n = 59) that collected data from a single country. Three additional articles, not shown in this figure, included data from multiple countries: one from Europe; one covering China, France, Germany, Italy, Japan, Spain, the UK, and the US; and one including data from the USA, South America, Northern Europe, Asia, and Africa.

As shown in Figure 2, the majority of articles (n = 58) reported data collected in a single country, mainly from high-income countries. Seventeen articles focused exclusively on data from the US, while 25 reported data from a single European country. Italy contributed the most articles (n = 8), followed by France (n = 4) and Germany (n = 3). Twelve articles included data from a middle-income country, with Kenya accounting for six of them, whereas only one article reported on data collected in a low-income country. Additionally, three articles reported data collected in multiple countries. As for the time of publication, most articles were published starting in 2023 (n = 45), while about half of the articles included in this review were published in 2024 alone (n = 30). The majority of studies used quantitative methods, with cross-sectional being the most frequent design, mainly using surveys for quantitative research and semi-structured interviews for qualitative research.

Regarding the type of prevention discussed, 10 articles examined attitudes towards RSV vaccines for elderly people and adults at risk (see Table 1), 24 regarded attitudes towards maternal RSV vaccines, of which 8 examined also attitudes towards infant immunization (see Table 2), and 27 focused on attitudes towards RSV immunization for infants, of which 16 mainly considered palivizumab (see Section 3.3). The main characteristics of the articles included are reported in the next sections, one for each group of articles (see the Supplementary Material for narrative summaries of all articles by group).

### 3.1. Knowledge and Attitudes Regarding RSV Vaccines for Elderly and Adults at Risk

Attitudes towards RSV vaccination for adults aged 60 years and older, as well as for individuals at risk, were assessed in 10 studies (Table 1) [30,31,32,33,34,35,36,37,38,39]. Four focused on healthcare professionals’ perceptions, with one using mixed (quantitative and qualitative) methods [31] and the rest using quantitative methods, including one focusing on primary care physicians [33], one focusing on different healthcare professionals (HCPs) [36], and one focusing on cardiac HCPs [37]. Among the six remaining articles on potential users of vaccines, one examined the general public [39], four examined adults over 60 years of age [30,32,35,38], and one focused on adults over 60 and younger adults with one or more chronic conditions [34].

**Table 1:** Characteristics of articles on knowledge and attitudes regarding RSV vaccines for elderly people and adults at risk.

Article	Aim	Country and Time	Methods and Sample	Main Results	Strengths and Limitations
Black 2023 [30] ^1,2^	To examine coverage of COVID-19, influenza, and RSV vaccines, and vaccination intent and sociodemographic characteristics	USA 24 September–9 December 2023	Quantitative cross-sectional survey. N = 62,816 adults >60 years of age	Estimated RSV vaccination coverage: 17.0% (21.4% among those with chronic health conditions). Throughout the study period, from 20.9% to 14.1% definitely intend to vaccinate for RSV. Relevant sociodemographic factors: insurance, age, urbanization, racial/ethnic group, jurisdiction.	Strengths: Very large sample size; data weighted to mitigate sample selection bias. Limitations: Low response rate; sample self-selection bias; self-reported measures; ad hoc non-standardized survey.
Ciemins 2023 [31]	To understand clinician knowledge, attitudes, behaviors, and beliefs around RSV and about the potential impact of a vaccine on the adult patient population	USA Not specified	Mixed, cross-sectional study. N = 74 clinicians (survey, n = 30; roundtable discussions, n = 7; interviews, n = 37)	RSV knowledge: average correct score, 56%; knowledge gaps: infection duration, comorbidities, risk factors. 57% of clinicians rarely or never see RSV patients; 40% have administered a health system test for RSV; 63% have no clinical treatment pathway for the diagnosis of RSV. Roundtable: participants agreed that more education about adult RSV would be beneficial.	Strengths: Assessment of clinicians; mixed qualitative and quantitative methods. Limitations: No specifics of different vaccines and settings; selection bias; small sample size; ad hoc non-standardized instruments; funded by pharmaceutical company.
Haeder 2024 [32] ^1^	To query Americans about their RSV vaccination status and their intention to get vaccinated this fall and winter	USA 27–28 September 2023	Quantitative cross-sectional survey. N = 1345 adults >60 years of age (representative sample)	Estimated RSV vaccination coverage: 9.1%. Vaccination intention: 42.2%. Correlates of intention to get vaccinated: levels of concern for the disease, self-assessed risks for the disease; belief that vaccines were safe and important; trust in health institutions. Correlates of hesitation to get vaccinated: not seeing the necessity for the vaccine, a lack of information, concerns about side effects, and concerns about vaccine safety.	Strengths: Large sample size; nationally representative sample of older adults. Limitations: Self-reported measures; ad hoc non-standardized survey.
Hurley 2019 [33]	To understand physicians’ knowledge, attitudes, and beliefs regarding RSV and a potential RSV vaccine	USA Febuary–March 2017	Quantitative cross-sectional study. N = 317 general internist and family physicians (≥50% in primary care and treated RSV patients in previous year)	86% would like more information about the burden of RSV in adult patients. 74% stated that influenza is generally more severe than RSV among patients aged over 50 years. 57% rarely consider RSV as a potential pathogen in patients 50 years or older with a respiratory disease. 61% do not test for RSV as there is no treatment. Anticipated barriers to RSV vaccination were financial.	Strengths: Assessment of primary care physicians; excellent response rate across the nation. Limitations:; self-report measures; limited validity (vaccine not offered; many physicians reported not having cared for a person with RSV); ad hoc non-standardized survey.
La 2024 [34]	To evaluate RSV-related knowledge, attitudes, and perceptions (KAP) among US adults at increased risk of severe RSV infection.	USA May–June 2022	Quantitative cross-sectional study. N = 827 adults (n = 224 aged 60–89 years) and patients at increased risk (18–59 years, n = 200 with a chronic cardiovascular condition; n = 347 with a chronic pulmonary condition; n = 308 with diabetes mellitus)	43.3% had previously heard of RSV (only 32.1% in adults aged 60–89). Of those aware of RSV, only 34.6% reported being knowledgeable about it (with only 12.9% answering correctly to questions about it), 33.7% reported worrying about it, and 67.3% rarely considered RSV as a potential cause of their cold/flu-like symptoms. Significant predictors of RSV awareness: awareness of other respiratory infections, having child(ren) in the household, being from Midwest geographic region, knowledge of respiratory infections, being female.	Strengths: Focus on at risk population eligible for vaccination. Limitations: Selection bias; self-reported measures; interdependence of data (some participants qualified for multiple groups due to a combination of conditions); ad hoc non-standardized survey.
Motta 2025 [35] ^1^	To study intentions to vaccinate against RSV, as well as potential barriers to vaccination, in a representative sample of adults aged 60 or older.	USA 20 October–6 November 2023	Quantitative cross-sectional study. N = 362 adults >60 years of age (representative sample)	Estimated RSV vaccine coverage: 14% In the process of receiving vaccination: 5% May vaccinate in the future: 27% Intend to refuse vaccination: 53% Correlates of vaccination intention: positive beliefs about vaccine safety and efficacy, prior influenza and COVID-19 vaccinations.	Strengths: Nationally representative sample of older adults. Limitations: Self-reported measures; ad hoc non-standardized instruments.
Papagiannis 2024 [36]	To assess the knowledge, attitudes, and anticipated vaccination practices among health professionals in Central Greece in response to the potential introduction of new RSV vaccination guidelines by the National Vaccines Committee	Greece 1 Octoner–31 December 2023	Quantitative cross-sectional study. N = 219 HCPs (pediatricians, pulmonologists, obstetricians, general practitioners, pharmacists, and nurses)	70.3% accurately identified the RSV vaccine’s current availability, and 62.1% confirmed the current recommendation for pregnant women, but 52.1% indicated that vaccination was available for infants up to 6 months, children, and adolescents. 97.3% adhere strictly to the vaccination recommendations provided by the National Vaccination Program. 93.1% would support RSV vaccination if it was available and endorsed by the National Immunization Program.	Strengths: Assessment of health professionals from diverse backgrounds. Limitations: Selection bias; small sample size; limited generalizability; limited validity (questions about potential and not actual introduction of new RSV vaccination guidelines); ad hoc non-standardized instruments.
Ponticelli 2024 [37]	To investigate the knowledge about and attitude towards RSV and RSV vaccines and the intention to recommend vaccination within a cardiological hospital in Italy.	Italy November 2023	Quantitative cross-sectional study. N = 154 HCPs (cardiologists and cardiac nurses)	46.9% were aware of market authorization of RSV vaccines for adults ≥60 years of age (predicted by older age, higher level of education, and having participated in a professional update about vaccination). >90% need more information about vaccination in older adults and chronic patients and about the RSV vaccine. 70.5% were willing to recommend/suggest RSV vaccination to patients.	Strengths: Assessment of actual knowledge of RSV vaccines; assessment of health professionals from diverse backgrounds. Limitations: Social-desirability bias; single-center study.
Vascimini 2024 [38] ^1^	To assess patient knowledge about RSV and to provide patients with information about the virus and vaccination for adults over 60 years of age.	USA 12 September 2023–11 March 2024	Quantitative cross-sectional study. N = 518 adults >60 years of age	78% had not received any education from their healthcare provider about RSV. 58–70% gave correct answers to RSV knowledge questions. 63% consented to receiving the vaccine on the day of the survey. Reasons for vaccine refusal: recent vaccination, lack of perceived necessity for vaccination, and concerns about potential side effects.	Strengths: Focus on source of information. Limitations: Selection bias; ad hoc non-standardized questions.
Wang 2024 [39]	To assess the public’s perceptions of respiratory syncytial virus (RSV) and attitudes toward the RSV vaccine and to identify associated factors in China.	China 16 August and 14 September 2023	Quantitative cross-sectional study. N = 2133 members of the general public (n = 115 >50 years of age)	24.3% had never heard of RSV, 44.3% believed that they were at risk of contracting RSV, and 59.8% perceived RSV infection to be somewhat serious. 68.4% were willing to receive the vaccine Adults aged >50 years had more frequently never heard of RSV (36.5%) and had a lower level of knowledge of RSV (55.3%), while also showing lower willingness to accept vaccination. Main facilitators of acceptance: worrying about RSV infection, wanting to protect people around you.	Strengths: Large sample size; perspective from low/middle-income countries. Limitations: Selection bias; limited validity (vaccine not offered); high proportion of high education level and economic status and very low proportion of older adults; ad hoc non-standardized survey.

^1^ Vaccine(s) already available: Black 2023 [30], Header 2024 [31], Motta 2025 [35], Vascimini 2024 [38]. ^2^ Also other illnesses: Black 2023 [30].

Overall, the findings on vaccination for adults showed that most samples have a rather low awareness of RSV and its prevention, but their attitude towards vaccination is frequently positive, especially when it is seen as a way to protect against a disease that is perceived as severe and when it is perceived as safe and effective. Specifically, awareness of RSV remained limited in the general public, adults over 60, and those at increased risk of complications, with an estimated 65% of respondents having never heard of RSV or only being familiar with the name [34,39]. Nevertheless, their attitudes were often positive, with vaccination intention and/or actual uptake ranging from 31% [30] to 68% [39] (see Table S2 for a summary of values of awareness and acceptance). Key facilitators of positive attitudes included concerns about the disease’s risks, the desire to protect vulnerable individuals, and trust in health institutions and vaccine safety [32,39]. Barriers included a perceived lack of necessity, insufficient information, concerns about vaccine safety and potential side effects, and apprehension about its novelty [32,39].

HCPs showed somewhat limited knowledge about RSV and RSV prevention in adults. Indeed, they frequently indicated a desire for more information about RSV, with rates ranging from 35% [36] to 87% [33] and 90% [37]. While some HCPs anticipated that economic issues would play an important role [33], others expected that COVID-19-related vaccine hesitancy would persist, with safety concerns being the main reason for reluctance to vaccinate [31,37]. HCPs expected the uptake of the RSV vaccine in adults to be comparable to that of the influenza vaccine [31].

Data collected from a very large sample during the initial rollout of the vaccination in the USA revealed lower rates of uptake and intention to get vaccinated. RSV vaccine uptake was 17%, with an intention rate of 14.1%, whereas influenza vaccine uptake was higher, at 69.3%, with an intention to get vaccinated of 8.2% [30]. Other data from a smaller sample suggested a higher proportion of individuals who were either vaccinated (9%) or who intended to get vaccinated against RSV (42%) [32]. Similarly, in a representative sample of the population, 14% were already vaccinated, 5% had booked a vaccination, and 27% planned to get it in the future [35]. A substantially higher uptake rate was found in a study conducted in community pharmacies, where the RSV vaccine was offered on site, and the acceptance rate was 63% [38]. The only non-US study based on acceptance in the population was conducted in China, with a convenience sample of adults, with a small proportion of older adults (5.4% over 50 years of age) [39]. While approximately 65% of participants had never heard of RSV or only knew the name, only one-third of participants expressed reluctance to get vaccinated.

### 3.2. Knowledge and Attitudes Regarding Maternal RSV Vaccines

The topic of maternal vaccines was examined in 24 articles, with 16 exclusively examining attitudes towards vaccination during pregnancy and 8 also examining attitudes towards maternal vaccines against RSV and antibodies for immunizing infants (Table 2). Three of these articles gathered data exclusively from healthcare providers [40,41,42], while two included input from both healthcare providers and pregnant women [43,44]. Most of the remaining articles focused on pregnant women, with some collecting data solely from this group [45,46,47,48,49,50,51,52,53,54] or also including lactating women [55,56,57], women planning pregnancy [58], recently pregnant women [59], or parents [60,61]. One study involved politicians [62]. One study examined the attitudes of young females to develop and validate a tool for assessing attitudes towards RSV vaccination [63].

**Table 2:** Characteristics of articles on attitudes regarding maternal RSV vaccines.

Article	Aim	Country and Time	Methods and Sample	Main Results	Strengths and Limitations
Adhikari 2024 [45] *	To explore knowledge of RSV, practices and knowledge regarding vaccination during pregnancy, and the willingness to accept vaccines against RSV during pregnancy in the future among mothers	Nepal 15 October–30 November 2023	Quantitative cross-sectional study. N = 340 pregnant women	98% had not heard about RSV, whereas only 4% had not heard of bronchiolitis (but 83% only knew the name). If an RSV vaccine was part of the national immunization schedule, 42% would definitely use it. 72% would prefer maternal vaccination, while 28% would prefer to vaccinate their children if such an option existed. All but one participant were willing to receive additional antenatal vaccines if approved for use in future.	Strengths: Perspective from low/middle-income countries; comparative analysis of vaccination targets (mothers vs. children). Limitations: Sample self-selection bias; self-reported measures; limited validity (vaccine not offered); ad hoc non-standardized survey.
Beusterien 2024 [43] *	To assess the impact of RSV preventive characteristics on the intentions of pregnant people and HCPs to protect infants with a maternal vaccine or monoclonal antibodies.	USA October–November 2022	Quantitative cross-sectional study. N = 1302 individuals: 992 pregnant people, 310 HCPs	In the discrete choice experiment, a preventive option (vs. none) was chosen 89.2% (pregnant people) and 96.0% (HCPs) of the time. Effectiveness was the most important attribute to preventive choice for both pregnant people and HCPs. All else equal, pregnant people and HCPs preferred a maternal vaccine over monoclonal antibodies (but this had limited influence on the choice). In best–worst scaling (BSW), longer protection, protection starting at birth or the beginning of the RSV season, and use for both pre-term and full-term babies were ranked highest in importance.	Strengths: Large sample size; use of a discrete choice test; comparative analysis of vaccination targets (mothers vs. children). Limitations: Sample self-selection bias; self-reported measures; limited validity (vaccine not offered); ad hoc non-standardized survey.
Cubizolles 2023 [46] ^2^	To assess intentions to get vaccinated against seasonal influenza, COVID-19, pertussis, and RSV in pregnant women and to identify factors associated with intentions	France Febuary–May 2023	Quantitative cross-sectional study. N = 310 pregnant women	Intention to get vaccinated during pregnancy: 39.4% Confidence in vaccines using the 5C-model [64] was associated with RSV vaccine acceptance, together with receipt of pertussis vaccination in the past 5 years and knowledge score.	Strengths: Employment of an established model of vaccine hesitancy. Limitations: Sample self-selection bias; limited validity (vaccine not offered); self-reported measures; ad hoc non-standardized survey.
Damatopoulou 2024 [47]	To evaluate pregnant women’s willingness to receive the RSV vaccine during pregnancy and factors associated with it	Greece April–December 2023	Quantitative cross-sectional study. N = 335 pregnant women	Limited awareness of RSV: 75.5% were unfamiliar with the infection and only 26% reported good knowledge of bronchiolitis. 81.7% were unwilling to receive RSV vaccine if in a trial and 55.2% if the vaccine was approved and recommended. Higher vaccine acceptance associated with higher education, having school-aged children, RSV awareness, willingness to vaccinate with routine pregnancy vaccines, and prior COVID-19 vaccination.	Strengths: Participants from diverse healthcare settings. Limitations: Sample self-selection bias; self-reported measures; limited validity (vaccine not offered); ad hoc non-standardized survey.
Gagneux-Brunon 2022 [40]	To explore attitudes of midwives about pregnant individuals’ participation in a vaccine clinical trial, as the promotion of clinical trials by midwives may facilitate the decision-making of pregnant individuals	France 11 September–11 November 2020	Quantitative cross-sectional study. N = 398 midwives	28.3% were willing to encourage pregnant individuals to participate in a hypothetical respiratory syncytial virus (RSV) vaccine clinical trial. Considering themselves to have good training about vaccines predicted promotion of the clinical trial. Vaccine hesitancy and psychological antecedents of vaccinations were not associated with a lower promotion of pregnant individuals trial participation by midwives.	Strengths: Implications for research clinical trials implementations. Limitations: Sample self-selection bias; limited validity (vaccine not offered); self-reported measures; ad hoc non-standardized survey.
Giles 2019 [48] ^2^	To survey pregnant women from diverse cultural backgrounds living in Australia to explore what they know about group B streptococcus and RSV and their attitudes regarding future vaccination	Australia November 2017–June 2018	Quantitative cross-sectional study. N = 495 pregnant women	83% had never heard of RSV. Factors influencing knowledge: age (over 35 years), nationality (Australia), number of children. 77% would be very likely to receive vaccination, 18% would be likely to receive vaccination, 3% would be unlikely, and 2% would be very unlikely Factors influencing vaccination intention: having received influenza or pertussis vaccine in pregnancy.	Strengths: Focus on factors influencing awareness. Limitations: Sample self-selection bias; limited validity (vaccine not offered); self-reported measures; ad hoc non-standardized survey.
Harteveld 2024 [49] *	To investigate the perception and willingness of pregnant women and their partners to accept maternal vaccination or neonatal immunization against RSV.	Netherlands Febuary–April 2024	Quantitative cross-sectional study. N = 1001 pregnant women and their partners	41% of participants had heard of RSV and 46% knew a lot about it. Acceptance for sure of RSV prevention was: 67% for a maternal vaccine and 64% for infant immunization, with a large overlap (56% willing to accept both for sure and an additional 31% leaning towards acceptance of both). Preference for the type of prevention: 75% of pregnant women preferred maternal vaccination alone and 8% combined with infant immunization, whereas only 3% preferred infant immunization only. Main reasons for acceptance: desire to protect the child (87%) and concerns regarding the severity of RSV infection (75%). Main reasons for refusal or hesitancy: lack of RSV knowledge (43–56%) and fear for the unborn child’s safety (23–34%).	Strengths: Large sample size; comparison of vaccination targets (mothers vs. children); perspective of both parents. Limitations: Sample self-selection bias; self-reported measures; limited validity (vaccine not offered); ad hoc non-standardized survey.
Holland 2024 [60] *	To assess parental awareness of RSV and the level of acceptance of future RSV prevention strategies.	Australia 15 July–27 August 2022	Quantitative cross-sectional study. N = 1992 parents	89.6% of current parents and 78.7% of future parents had heard of RSV. Of those, between 50.0 and 64.2% were aware of its association with pneumonia and between 52.1% and 71.8% were aware of its association with bronchiolitis. Factors associated with higher RSV awareness: Australian-born parents, living in the eastern states, with a university-level education, and being a current parent. High level of acceptance for maternal vaccines (future parents: 79.3%) and infant immunization (all: 81.7%).	Strengths: Large sample size; comparison of vaccination targets (mothers vs. children); perspectives of both parents. Limitations: Self-reported measures; limited validity (vaccine not offered); ad hoc non-standardized survey.
Kherfan 2023 [63]	To assess the acceptance of the RSV vaccine among young females and identify the determinants influencing their decision; to develop and validate a survey instrument to measure RSV vaccine acceptance	Jordan 5–6 July 2023	Quantitative cross-sectional study. N = 315 young females	67.6% had heard of RSV before the study. 70.2% would be willing to get the vaccine, 15.2% would refuse, and 14.6% were hesitant. Development and validation of a 21-item RSV vaccine acceptance scale “ABCDEF”, with six subscales: Advice, Burden, Conspiracy, Dangers, Efficiency, and Fear. RSV vaccine acceptance was associated with the “advice” and “fear” factors.	Strengths: Develops and validates a questionnaire; perspective from low/middle-income countries. Limitations: Sample self-selection bias; limited validity (vaccine not offered); self-reported measures.
Limaye 2023 [55]	To understand factors that could inform maternal vaccine decision-making to inform future demand generation strategies in Kenya	Kenya August–September 2022	Qualitative cross-sectional study. N = 24 pregnant and lactating women	Factors influencing decision to vaccinate: awareness of RSV; risk perception and causes of RSV. Key participants’ questions were related to vaccine side effects, vaccine effectiveness, as well as timing of administration and dosing.	Strengths: Perspective from low/middle-income countries. Limitations: Sample self-selection bias; small sample size; limited validity (vaccine not offered).
Limaye 2023 [56]	To examine attitudes toward maternal RSV vaccines among pregnant and lactating persons in Kenya	Kenya Fall 2022	Quantitative cross-sectional study. N = 400 pregnant and lactating women (+ 18 years of age, lactating or II/III trimester)	87% perceived RSV prevalence as high, 54% perceived RSV risk as high, 80% perceived supportive norms as high, 87% perceived themselves as having high self-efficacy, 83% had low perceived barriers, 97% had high confidence in vaccine safety, and 89% had high trust in vaccine effectiveness. Factors influencing vaccine hesitancy among both pregnant people and lactating people: primigravida more hesitant than multigravida. For lactating people, lower vs. higher social norms of RSV vaccine acceptance were associated with higher hesitancy.	Strengths: Employment of an established model of drivers of vaccine uptake; perspective from low/middle-income countries. Limitations: Sample self-selection bias; self-reported measures; limited validity (vaccine not offered); ad hoc non-standardized survey.
Limaye 2024 [41]	To better understand how lessons learned from the COVID-19 vaccine rollout among pregnant and lactating people in Kenya could inform a future maternal RSV vaccine rollout.	Kenya August–September 2022	Qualitative cross-sectional study. N = 16 healthcare providers (doctors, nurses, midwives, community health workers, and vaccinators)	Community sensitization emerged as the most critical lesson learned, including three domains: (a) communication (ensure community awareness of RSV and its harms and the benefits of RSV maternal vaccines, and providing up-to-date, clear information about maternal RSV vaccines); (b) mobilization (healthcare providers and community leaders need to gain the trust of communities, and the importance of routinizing the vaccine), and (c) education (critical questions related to vaccine safety concerns, duration of protection, and vaccine dosing).	Strengths: Perspective from low/middle-income countries; linking RSV and COVID-19 vaccine experience. Limitations: Sample self-selection bias; small sample size; limited validity (vaccine not offered).
Limaye 2024 [62] ^2^	To examine the fundamental factors determining good adoption and adherence to RSV vaccines in pregnant women, with contributions from some political leaders	Kenya August–September 2022	Qualitative cross-sectional study. N = 20 politicians	The policy process for new maternal vaccine introduction requires substantial evidence and coordination among diverse stakeholders, and it goes beyond the adoption of a new policy. Need to allocate adequate resources for the education of communities given the sensitive target group (pregnant women).	Strengths: Perspective from low/middle-income countries; practical implications for vaccine policy. Limitations: Sample self-selection bias; small sample size; limited validity (vaccine not offered).
Maculaitis 2024 [50] *	To identify subgroups of pregnant people with similar preferences and priorities for infant immunization and to identify the characteristics of each subgroup and how they vary across subgroups (secondary analysis of data from Beusterien [65])	USA 11 October–11 November 2022	Quantitative cross-sectional study. N = 992 pregnant people	Latent class analysis identified three preference subgroups: ‘Effectiveness’ (preventive choice mostly driven by increases in effectiveness; 51.4%); ‘Season’ (preventive choice mostly driven by improvement in duration of protection during the RSV season; 39.2%); and ‘No Preventive’ (frequently chose no-preventive option; 9.4%). ‘Effectiveness’ and ‘Season’ preferred maternal vaccine over monoclonal antibodies, and vice versa for ‘No Preventive’. Perceiving RSV as serious for infants, higher health literacy, and lower household income were associated with ‘Effectiveness’. Perceiving RSV as serious for pregnant people was associated with ‘Season’. Perceiving RSV as not being serious for pregnant people and not being employed were associated with ‘No Preventive’.	Strengths: Large sample size; identification of determinants of vaccination targets (mothers vs. children). Limitations: Sample self-selection bias; self-reported measures; limited validity (vaccine not offered); ad hoc non-standardized survey.
McCormack 2024 [51]	To examine maternal RSV awareness, likely acceptance of RSV vaccination in pregnancy, and attitudes to maternal vaccination	Ireland December 2018–April 2019	Quantitative cross-sectional study. N = 528 pregnant women	75.6% had never heard of RSV. 48.5% would avail of a vaccine, 45.8% were undecided, and 5.3% would not. The main reason (76.4%) for accepting vaccination was that it protects the infant from illness. The general practitioner (GP) was the preferred guidance source in decision-making (57.7%).	Strengths: Focus on critical role of trusted sources in guiding vaccination decision-making. Limitations: Sample self-selection bias; self-reported measures; limited validity (vaccine not offered); ad hoc non-standardized survey.
Miraglia del Giudice 2023 [52] *	To evaluate pregnant women’s awareness regarding RSV infection and willingness to receive the vaccine during pregnancy and to vaccinate their newborn against RSV	Italy 20 April–30 June 2023	Quantitative cross-sectional study. N = 490 pregnant women	32.2% have heard about RSV and 64.7% would like additional information about prevention strategies against RSV. After being provided information, 20% of pregnant women were very concerned about RSV infection, 16% considered maternal vaccination very useful, and 22% considered infant immunization very useful. 45.9% were willing to undergo maternal vaccination and 61.1% were willing to immunize the newborn. Protecting the child was the main reason to accept vaccination (71% for a maternal vaccine and 76% for infant immunization), while the main reason to refuse was being concerned about side effects (51% for a maternal vaccine and 42% for infant immunization).	Strengths: Focus on comparative analysis of vaccination targets (mother vs. children). Limitations: Sample self-selection bias; self-reported measures; ad hoc non-standardized survey: limited validity (vaccine not offered)
Nyawanda 2023 [42]	To assess the current knowledge, attitudes, and perceptions around RSV disease and RSV prevention products in development among HCPs in Kenya	Kenya September–October 2021	Mixed cross-sectional study. N = 106 healthcare workers	39.4% had heard about RSV, only 2% were aware of RSV prevention products that were either available or in development. 92.7% felt that pregnant women should be vaccinated against RSV. Between 50.0% and 61.3% would recommend a single-dose vaccine schedule for maximal adherence and compliance or to prevent wastage and contamination, and to provide maternal vaccination through antenatal care clinics.	Strengths: Mixed qualitative and quantitative methods; perspective from low/middle-income countries. Limitations: Sample self-selection bias; self-reported measures; limited validity (vaccine not offered); ad hoc non-standardized survey.
Paulson 2024 [61] *	To explore parents’ views regarding the different methods of protecting infants against RSV. Understanding factors associated with acceptance or hesitance is essential to implement a successful vaccine program	UK August–September 2023	Quantitative cross-sectional study. N = 1620 parents <2 years and/or pregnant people	11% had never heard of RSV and 19% were unfamiliar with it (lower knowledge than bronchiolitis, pneumonia, and flu). 88% would accept maternal vaccination, while 78–79% would accept infant monoclonal antibodies Most important reasons for acceptance: desire to protect infant, knowledge about safety and about effectiveness. Most common reasons for hesitancy: safety, lack of knowledge about the vaccine and the antibodies, concerns about duration of protection, concern about the number of vaccines given to infants. 82.6% expressed a preference for a vaccine in pregnancy over infant antibodies. Currently being pregnant was significantly associated with a preference for a maternal vaccine, whilst not receiving routinely recommended antenatal vaccines was associated with a preference for infant antibodies.	Strengths: Multi-center study; high response rate: large sample size; identification of determinants of vaccination targets (mothers vs. children). Limitations: Sample self- selection bias; self-reported measures; ad hoc non-standardized survey.
Sallam 2024 [53]	To assess the willingness of pregnant women in Jordan to receive RSV vaccination and its associated determinants.	Jordan January–Febuary 2024	Qualitative cross-sectional study. N = 404 pregnant women	If a vaccine was proven safe and effective and it was free, 77.5% would be willing to receive RSV vaccination, 6.2% would be hesitant, and 16.3% would be resistant. Predictors of higher acceptance were: age < 30, undergraduate education, being HCPs, higher income, having received at least 3 doses of COVID-19 and flu vaccines. On the ABCDEF validated scale, constructs positively associated with acceptance included Advice, Burden, Efficiency, and Fear (of infection), whereas Conspiracy and Danger were negatively associated with acceptance.	Strengths: Use of validated scale; multi-center study; perspective from low/middle-income countries. Limitations: Sample self-selection bias; self-reported measures; limited validity (vaccine not offered); ad hoc non-standardized measures.
Saper 2024 [58]	To characterize interest in RSV vaccination during pregnancy among people across the United States who were pregnant or planning to become pregnant.	USA March 2023	Quantitative cross-sectional study. N = 1528 pregnant people or those planning pregnancy	20% had never heard of RSV. 40% perceived RSV illness in children as both serious and likely, 45% considered it serious but unlikely, and 16% did not view it as serious. 54% reported being very likely to receive an RSV vaccine during pregnancy. Stronger acceptance of RSV vaccination when they had previously received other maternal vaccinations.	Strengths: Large sample. Limitations: Sample self-selection bias; self-reported measures; limited validity (vaccine not offered); ad hoc non-standardized instruments.
Singh 2024 [57]	To explore key interpersonal influences on maternal vaccine decision-making among pregnant and lactating people and community members in Kenya	Kenya July–September 2022	Qualitative cross-sectional study. N = 34 people (6 pregnant, 18 lactating, and 10 community members)	79% stated that the pregnant person themself should be the primary decision-maker about maternal vaccination. 56% of all interviewees believed that HCPs were or should be involved in the maternal vaccination decision. 41% explicitly stated that male partners should not influence the process whereas 35% highlighted the importance of male partners being involved in the process and providing their approval before making a decision.	Strengths: Perspective from low/middle-income countries. Limitations: Sample self-selection bias; limited validity (vaccine not offered); small sample; ad hoc non-standardized procedures for data collection.
Strózik 2024 [59] ^1^	To assess RSV vaccination coverage among pregnant women, identify factors contributing to vaccination hesitancy, and evaluate the knowledge of the benefits of vaccination among patients in Poland	Poland 25 July to 1 August 2024	Quantitative cross-sectional study. N = 668 women who had recently given birth or were about to	Overall, only 2% of participants did not know what RSV is. Participants working in medical professions (22%) were more knowledgeable about RSV (assessed with 8 ad hoc questions); more likely to have been vaccinated against RSV (25.2%) than participants not in medical professions (12.9%), with an overall uptake of 15.6%; more likely to have been vaccinated for other diseases during pregnancy (23.3% vs. 1.7%); and more likely to have been informed by their physicians about RSV vaccination (25.3% vs. 9.6%).	Strengths: Report on actual uptake of RSV vaccination. Limitations: Sample self-selection bias; self-reported measures; ad hoc non-standardized survey.
Tucker 2024 [54] ^1,^*	To analyze the acceptance of the RSV vaccine among pregnant individuals in the first season of its offering	USA September 2023–January 2024	Quantitative cross-sectional study. N = 206 pregnant women	Out of the 206 patients who were eligible for the RSV vaccine, 53.8% were offered the vaccine. Of these, 55.9% accepted it and 44.1% declined. 71% of decliners consented to be interviewed. The main reasons to decline vaccination were distrust of new vaccines (80%), fear of side effects for the fetus (46%), including in the long-term (23%) and not limited to this vaccination in pregnancy (17%). They were more likely than acceptors to have declined other maternal vaccines. 94% of decliners reported that their clinician adequately discussed the vaccine but 34% felt that they had not had enough time to decide. 63% would accept newborn RSV antibodies and 66% would likely accept the vaccine in a future pregnancy.	Strengths: Validity (actual offer of vaccination); focus on decliners of RSV vaccination. Limitations: Sample selection bias; self-reported measures; ad hoc non-standardized measures.
Wilcox 2019 [44]	To determine (1) the awareness of RSV among pregnant women and healthcare professionals (HCPs) and (2) attitudes toward clinical trials and routine implementation of antenatal RSV vaccination	UK July 2017–January 2018	Quantitative cross-sectional study. N = 525 individuals (321 pregnant women and 204 maternity HCPs)	Most pregnant women (88%) and midwives (66%) had no/very little awareness of RSV, unlike obstetricians (14%). 29% of pregnant women would likely accept RSV vaccination as part of a trial, and 75% if routinely recommended. Factors associated with higher acceptance of vaccination: if in a clinical trial: younger age (16–24 years), 21–30 weeks’ gestation, experience of RS; if routinely recommended: White-British women, 21–30 weeks’ gestation. Obstetricians were more likely than midwives to support vaccination both if it was in a clinical trial and as part of routine RSV vaccination, as were those with prior knowledge of RSV and who deemed it serious.	Strengths: Implications for feasibility of clinical research trials. Limitations: Sample self-selection bias; self-reported measures; limited validity (vaccine not offered); ad hoc non-standardized survey.

^1^ Vaccine(s) already available: Strózik 2024 [59]; Tucker 2024 [54]; ^2^ also other illness(es): Cubizolles 2023 [46], Giles 2019 [48], Limaye 2024 [41]; * also attitudes towards RSV immunization for infants.

Regarding maternal vaccination, the share of participants who were unfamiliar with RSV varied greatly, ranging from 2% [59] to 98% [45], with five studies reporting less than 50% of participants being unaware of RSV [53,58,59,60,61], four reporting between 51% and 80% being unaware of RSV [47,49,51,52] and three reporting over 81% being unaware of RSV [44,45,48] (see Table S2). Nonetheless, they showed positive attitudes towards vaccinating during pregnancy if vaccines were offered as a routine vaccination, with values of intention to get vaccinated ranging from 42% [45] to 88% [61]. Data about actual coverage were scarce, with an estimated coverage of 15.6% in Poland (reported in a pre-print article) [59] and 56% among those who were offered it in the USA [54]. Additionally, between 18% [47] and 29% [44] expressed willingness to participate in a clinical trial on RSV vaccines. HCPs involved in maternal health also showed relatively low awareness of RSV and its prevention but held positive attitudes [42], including in the context of clinical trials [40,44]. For both HCPs and pregnant women, when comparing the effect of different attributes of preventative options, participants’ preferences were driven mainly by the effectiveness in preventing severe forms of illness, followed by the duration of protection [43].

Positive predictors of acceptance of RSV maternal vaccination included perceived protection, the perceived severity of RSV (especially for infants), confidence in vaccines, and having received other maternal vaccinations [46,48,49,51,52,58,59,61]. Concerns about the safety of the vaccine, especially for the baby, were the primary reason for refusal or hesitation about vaccination, along with a lack of RSV knowledge [46,52,61]. This was confirmed in one of the few studies assessing actual uptake of vaccination, when interviewing pregnant women who had refused maternal RSV vaccination, showing that the primary reason for refusal was distrust of new vaccines (80%), followed by fear of side effects for the fetus (46%) or the baby in the long term (23%) [54]. Finally, one article reported on the development and validation of an RSV vaccine acceptance scale [63]. Only two studies referred to models of vaccine acceptance or hesitancy, one [56] used the Behavioral and Social Drivers of Vaccination framework (BeSD) [66,67], the other one [46] employing the 5C model of vaccine hesitancy (comprising confidence, complacency, constraints, calculation, and collective responsibility) [68], and showed results in line with the literature [64,69].

One of the key choices that is already possible, or will be in the near future, is the choice between maternal vaccination and infant immunization. As summarized in Table 3, all but one study [52] examining attitudes towards both prevention strategies showed a preference for maternal vaccination over infant immunization, both when offered as single options and when the option of combining them is available. Moreover, the findings from the DCE study showed that the type of prevention had a small but significant effect on preferences, with a maternal vaccine preferred over monoclonal antibodies and pregnant women preferred over infants as the recipients of the injection [43]. Furthermore, when classifying participants based on their preferences using latent class analysis (LCA), pregnant women whose preferences were driven by the effectiveness or duration of protection favored maternal vaccination over infant immunization. In contrast, pregnant women who often opted for the no-prevention choice tended to prefer infant immunization over maternal vaccination [50]. In line with the latter finding, in the study focusing on people who refused maternal RSV vaccination, 63% declared that they would immunize their infant [54], and another study found that not receiving routinely recommended antenatal vaccines was associated with a preference for infant antibodies [51].

### 3.3. Attitudes Towards RSV Immunization for Infants

Attitudes towards using antibodies for immunizing infants against RSV were assessed in 27 articles (Table 4). Of these, nine examined attitudes in healthcare professionals [70,71,72,73,74,75,76,77], one in caregivers [78], and the rest examined them in parents or parents to be. Overall, 16 articles related to palivizumab, either in general [65,70,72,73,74,75,76,77,78,79] or specifically regarding compliance with it or its administration [80,81,82,83,84,85].

Among articles about palivizumab, seven focused on HCPs [70,72,73,74,75,76,77]. The findings generally highlight low awareness of RSV prophylaxis; for instance, only half of the participants were aware of it in one of the oldest studies [70], while several knowledge gaps were also found recently [74,75,76]. Nonetheless, attitudes towards RSV prevention were largely positive, with between 75% and 82% of pediatricians supporting the use of monoclonal antibodies and 92–94% being in favor of a potential future vaccine [71,75,76].

One study found that the information provided by medical practitioners to families regarding palivizumab was inaccurate, failing to correctly describe the effect of palivizumab on the severity of the illness, with it being described as if it reduced the need for ventilation and mortality [77]. Similarly, the information provided during counseling was found to drastically affect palivizumab administration in the Netherlands [79]. When it was described as recommended, the uptake rate was between 89 and 99%, whereas when it was described as a personal choice (preference-sensitive decision) and the benefits were described using the number needed to treat (NNT), specifying that 20 children need to be treated to prevent one hospitalization, the uptake was only 8%.

Since the effectiveness of palivizumab is short-term, compliance with the monthly schedule is essential for ensuring immunization. From the HCPs’ perspective [80], the most common barriers were caregiver inconvenience, distance to the clinic, cost of prophylaxis, and a lack of understanding of the severity of RSV. As interventions to improve compliance with palivizumab prophylaxis schedules, participants recommended the provision of educational materials about RSV, reminders from the hospital or clinic, and administration of prophylaxis at home to increase compliance. The latter was supported also by parents who participated in a pilot trial about at-home immunization [81]. All participants who received the immunization at home would prefer it there, and 70% of those in hospital would prefer to receive it at home. Different interventions to increase compliance were also tested, and a reminder two days early with a request to plan the appointment day was the most effective in increasing compliance (97% vs. 91% in the control group) [82].

The studies examining the perspectives of parents or parents to be on immunizing their children against RSV showed that between 26% [94] and 65% [92] had not heard of RSV or only know the term (see also Table S2), while intention to vaccinate varied from 38% [88] to 97% [95]. The only study reporting actual uptake was conducted in France, and the uptake was 92% [86].

The main reason for acceptance was the protection of the infant, although perceiving the illness as severe also contributed to acceptance [65,87,90,91,92,93,95]. The main reason for refusal was concerns about safety and side effects [65,91,92,93,95].

## 4. Discussion

Recent progress in RSV prevention strategies has attracted considerable attention and new prevention options are under development [15,16,17,18,19,20,21,22,23,24,25,26,27]. As for all immunization programs, the success of RSV prevention strategies depends upon the awareness about them and their acceptance by the (parents/caregivers of the) target population [96,97]. This systematic review gathered 61 articles reporting on knowledge and various attitudes, including beliefs, perceptions, preferences, opinions, views, acceptance, intention, and uptake, regarding RSV prevention.

Compared to the other topics, a relatively small number of articles focused on vaccination for the elderly and adults at risk (n = 10). This is likely due to the relatively recent appreciation of the burden of this disease among adults and seniors [97,98,99,100,101,102]. On the other hand, most articles examined attitudes towards the passive immunization of infants, with roughly half examining attitudes towards vaccination during pregnancy (n = 24, with a subset of 8 studies also examining preferences for maternal versus infant immunization) and the other half focusing on infant immunization (n = 27, of which 16 focused on the short-term monoclonal antibody palivizumab, with a subset of 6 studies assessing adherence to its monthly administration). Approximately one-third of the articles evaluated attitudes among healthcare professionals (n = 19), with half of these focusing on infant immunization.

A common finding in most articles examining potential recipients of RSV prevention was low awareness about RSV, with a significant proportion of participants either never having heard of it or only knowing the name (see Table S2). In contrast, awareness of bronchiolitis was generally more widespread, especially among parents, in line with the literature on patterns of online queries [103], although this may also depend on the use of the terms that are common in everyday language, for instance, in the news, and may also depend on the language. Despite this limited awareness, attitudes towards RSV prevention were generally often positive, with acceptance rates ranging from 16% to 97% (Table S2). The primary reasons for acceptance included the desire for protection against the disease and recognition of its severity, while concerns about safety and potential side effects were the most frequently cited reasons for refusal or hesitancy.

Specifically, data on the acceptance of RSV vaccination in adults shows generally rather low levels of acceptance, with the proportion of people already vaccinated ranging from 9 to 17% and an additional 8 to 42% intending to get vaccinated [30,32,35], with the exception of one study showing a 63% uptake in community pharmacies [38] and one hypothetical study on the general population over 18 years of age reporting that 68% would accept vaccination [39]. This discrepancy may be due to several factors, including limited vaccine availability during the initial rollout, cautious recommendations from trusted agencies and HCPs, the eligibility of different age groups, and heightened caution regarding the novelty of the vaccine. Moreover, the higher uptake rate in pharmacies suggests that practical issues such as the ease of access may also be relevant. Also, the Advisory Committee on Immunization Practices has changed the recommendation for RSV vaccination from all people ≥60 years using shared clinical decision-making (June 2023) to all adults aged ≥75 years and adults aged 60–74 years who are at increased risk for severe RSV (August 2024) [104].

Articles examining attitudes towards RSV prevention in infants generally confirmed a limited awareness of RSV and its prevention, whereas parents were more often aware of bronchiolitis, sometimes through direct experience, as confirmed also by infodemiology data [103]. Concerns about the severity of RSV infection were quite sparse, with severity being perceived to be lower in samples with older children. Nonetheless, attitudes toward vaccination were generally positive, with moderate to high acceptance of immunization. Interestingly, acceptance was also found to depend on how the benefits were conveyed: using the number needed to treat (e.g., 20 children need to be treated to prevent one hospitalization) and framing the decision as a preference-sensitive decision resulted in lower acceptance of immunization [79]. Additionally, HCPs generally showed very positive attitudes, with pediatricians being slightly more favorable toward active rather than passive immunization [71,75].

The findings of articles on maternal vaccination showed that pregnant people and people planning pregnancy were often unfamiliar with RSV but were often accepting of vaccination during pregnancy if it was recommended, although the acceptance rates varied between studies. Worrying about potential risks for the baby in the short and long term was the most frequent reason for refusing or being hesitant about vaccination [46,48,52,61]. In line with similar findings about COVID-19 maternal vaccination [64,69], some evidence indicates that this hesitation may be transient and, at least partially, rooted in the novelty of the vaccination. Indeed, about 80% of women who refused maternal RSV vaccination distrusted new vaccines, together with the fact that about one-third indicated that they did not have enough time to decide whether to take a maternal RSV vaccine, while about two-thirds responded that they would get vaccinated in a future pregnancy [54].

The positivity of attitudes towards prevention was found also in a study using DCE, where a majority of pregnant women (89%) and HCPs (96%) expressed a preference for any prevention over no prevention, regardless of the characteristics of the prevention options considered [43]. It is worth noting, however, that this study presented participants with a choice between three options: two preventative strategies and a no-prevention option. In decision-making literature, this could sway preferences in two different ways: if the two prevention options are too similar, the choice could become too difficult, leading people to prefer the no-prevention option (similarity decoy effect). Conversely, if one preventative option is clearly inferior to another (e.g., lower effectiveness and lower duration of protection), the superior option may be preferred over the no-prevention option more frequently when the inferior (decoy) option is present. Considering that several products are being developed to prevent RSV, it is possible that people will be able to choose between different options, which could further increase vaccine acceptance [105] if the options are not too similar.

It is worth noticing the great variability in the methodological approaches across studies, with most using ad hoc measures, complicating the comparison and synthesis of findings. For instance, knowledge about RSV was assessed with a variety of questions, with the most commonly used being about whether people were familiar with the term (awareness of RSV), while the other questions were quite diverse, with only some overlap between studies. Even when measures appear similar, they may differ in wording, capturing slightly different aspects of the intended construct, or offer varying response options, further limiting comparability. For example, when assessing vaccination intentions, the ‘unsure’ option was sometimes presented separately (e.g., on a seven-point Likert scale [39]), sometimes grouped with other responses (e.g.,, “probably will get vaccinated or unsure” [30]), or not present at all (e.g., only yes or no answers [70]). Additional issues emerge when measuring intentions during a vaccine rollout, as behaviors and intentions dynamically change overtime [30]. Furthermore, few articles referred to established models, with only one study [46] employing the 5C model of vaccine hesitancy (comprising confidence, complacency, constraints, calculation, and collective responsibility) [68] and another [56] using the Behavioral and Social Drivers of Vaccination framework (BeSD) [66,67].

Another important consideration is that most studies were conducted in high-income countries (see Figure 2), following the recent introduction of new RSV prevention options. While this limits the generalizability of the findings, it also highlights the importance of addressing global equity and supply. From a public health standpoint, the push for affordable and universal access to RSV vaccines is essential. Ensuring that both adult vaccination and infant immunization are integrated into routine immunization programs can contribute to more comprehensive protection and reduce the burden of RSV-related morbidity. Policies that support equitable distribution and funding models that prioritize affordability can help to achieve broader coverage, particularly in underserved communities where RSV poses significant risks. These efforts align with the broader goal of strengthening healthcare resilience and promoting universal coverage through preventive measures [96].

Current studies also highlight the pressing need for greater investment in education, both within healthcare settings and among the general population. Such investments are crucial for building a well-informed public that can make evidence-based decisions and embrace vaccination as a key preventive tool [106].

## 5. Conclusions

This review of quantitative and qualitative studies highlighted that, despite awareness and knowledge of RSV and its prevention being limited, attitudes towards prevention strategies are generally positive, with moderate to high rates of acceptance. The reasons guiding acceptance or refusal are similar for all RSV prevention strategies, with protection against the disease and the perceived severity of the disease promoting acceptance, while concerns about side effects leading to refusal or some level of hesitancy. As more options are likely to become available, preferences may be further affected by the options available, and it is likely that more options are going to be available and approved in the near future. The evidence suggests that the effectiveness and length of protection will play an important role in shaping preferences. Furthermore, all things being equal, maternal vaccination tends to be more acceptable than infant immunization.

Moreover, the evidence underscores the importance of addressing the knowledge gap about RSV, its risks, and the benefits and potential side effects of vaccination, both among the public and healthcare professionals. Education about RSV should be guided by decision-making research to ensure clear and effective communication in healthcare settings.

Given the role of HCPs as trusted sources of information, their knowledge and confidence in discussing RSV prevention are pivotal. Training programs designed to boost familiarity with RSV epidemiology, prevention strategies, and updated clinical trial results could foster more effective patient counseling. Enhanced training covering communication strategies to address vaccine safety concerns and counter misinformation can empower HCPs to engage in meaningful discussions, fostering informed, confident decision-making.

## Figures and Tables

**Table 3:** Percentage of participants who prefer maternal vaccination, infant immunization, or a combination of the two as RSV prevention strategies.

Article	Maternal Vaccination	Infant Immunization	Both
Adhikari 2024 [45]	72%	28%	NA
Paulson 2024 [61]	83%	17%	NA
Miraglia del Giudice 2023 [52]	46%	61%	NA
Harteveld 2024 [49]	75%	3%	8%
Holland 2024 [60]	39%	4%	49%

**Table 4:** Characteristics of articles on attitudes regarding RSV immunization for infants.

Article	Aim	Country and Time	Methods and Sample	Main Results	Strengths and Limitations
Al-Jaid 2023 [65] *	To assess parental knowledge of RSV infection and attitudes regarding infant immunization with monoclonal antibodies	Saudi Arabia Febuary–June 2023	Quantitative cross-sectional study. N = 606 parents	48.7% had never heard of RSV, 20.1% only knew a little bit about it. 50.2% would accept RSV immunization if recommended, and 48.8% would proactively ask an HCP about it. Most commonly reported reason for acceptance: “to protect infant at an age where they are most susceptible to RSV” (45.5%). The most commonly cited important information about RSV vaccination was safety (75%), followed by efficacy (39.8%), while the least reported was cost (11.4%). Most commonly reported reasons for refusal was lack of knowledge regarding the immunization (22.3%), followed by lack of knowledge regarding RSV (9.7%) and concern and worry about effectiveness of RSV immunization (8.6%).	Strengths: It includes perspective of both parents. Limitations: Sample self-selection bias; self-reported measures; limited validity (vaccine not offered); ad hoc non-standardized survey.
Alvaro 2000 [70] *	To determine the opinions of pediatricians on the geographical and seasonal distribution and the clinical impact of RSV in Italy	Italy May–June 1999	N = 344 pediatricians	66.3% of the pediatricians interviewed had admitted children affected by RSV into their own department. For the participants, the most affected newborn babies were those of lowest gestational age, although 26.4% of admissions were full-term babies. Only half of the pediatricians were aware of the possibility of administering palivizumab at the time of the study (1999).	Strengths: Multi-center study. Limitations: Sample self-selection bias; self-reported measures; ad hoc non-standardized survey.
Anderson 2009 [80] *	To identify barriers encountered towards vaccination and advice on how improve adherence	Global Time not specified	Qualitative cross-sectional study. N = 100 doctors	The primary barriers to adherence to the full dosing schedule of palivizumab were inconvenience to parents and distance to the clinic, cost of palivizumab, and a lack of understanding of the threat of RSV. 68% believed that their patients’ parents think that palivizumab is a vaccine, and 38% of the responding physicians also believed that palivizumab is a vaccine. 86% of the respondents provide information to their patients’ caregivers about RSV and prophylaxis.	Strengths: Multi-nation study. Limitations: Low response rate; sample self-selection bias; self-reported measures; ad hoc non-standardized survey.
Congedo 2024 [71]	To investigate Italian pediatricians’ knowledge, attitudes and behaviors towards RSV and its prevention	Italy Febuary–May 2023	Quantitative cross-sectional study. N = 507 pediatricians	98% of pediatricians reported managing bronchiolitis cases, while only 44% had prescribed monoclonal antibodies. Knowledge of RSV was rather poor, with 36% unaware that passive immunization to prevent RSV is available for infants under two years of age and 25% mistakenly believing that active immunization was currently available. Attitudes toward RSV prevention: (a) 92% would support the use of a vaccine against RSV; (b) 82% would support the administration of monoclonal antibodies to all newborns and children during their first RSV season; and (c) 92% would support the implementation of an RSV prevention strategy for all newborns and children to help prevent complications such as bronchospasm and asthma.	Strengths: National study. Limitations: Low response rate; sample self-selection bias; self-reported measures; ad hoc non-standardized survey.
de Sentuary 2025 [86]	To evaluate the acceptance and safety of the treatment of newborns with nirsevimab during the first season of implementation	France 18 September 2023–23 January 2024	Quantitative cross-sectional study. N = 477 parents of newborns	The acceptance rate for nirsevimab was 91.6%. Factors such as the mother’s age, lower parity, and having a partner in work were significantly associated with higher acceptance. The main reason for accepting the treatment was to “protect my baby”, while the most common reason for refusal was the lack of long-term data on nirsevimab.	Strengths: Longitudinal prospective design; perspective of both parents; identification of interpersonal determinants of vaccination decision. Limitations: Low response rate at follow up; sample self-selection bias; self-reported measures; ad hoc non-standardized survey.
Ebersjö 2023 [81] *	To evaluate safety aspects and explore parents’ preferences regarding home versus hospital immunization with palivizumab during one RSV season	Sweden time not specified (IRB approval 2013)	Mixed quantitative and qualitative, with a pilot RCT. N = 43 parents	Most parents would prefer immunization with palivizumab at home if they were allowed to choose (100% of those receiving it at home and 70% of those receiving it in hospital). The content analysis of the reasons for their preference revealed three topics around (1) the protection and monitoring of the infant, (2) the health and well-being of the family, and (3) the avoidance of suffering for the infant. Importance of involving parents who previously experienced neonatal intensive care in the choice of place of immunization.	Strengths: Mixed qualitative and quantitative methods; controlled trial. Limitations: Sample self-selection bias; self-reported measures; ad hoc non-standardized measures.
Erolu 2024 [82] *	To analyze the effectiveness of two nudge interventions in increasing adherence in children with chronic heart disease	Turkey October 2020–April 2021	Quantitative cross-sectional RCT. N = 229 parents	Relative to the control group (adherence 90.9%), the first intervention (default bias, implementation intention) significantly increased adherence (97.3%), whereas the second intervention did not (availability bias, social norms, 94.2%). When only considerin children in their first RSV season, both interventions increased adherence (default bias, implementing intention 97.7%; availability bias, social norms 97.1%) compared to the control group (88.3%). Predictors of higher adherence: father employed and having an additional child.	Strengths: Theoretical background on the established literature on cognitive biases; prospective study; perspective from low/middle-income countries. Limitations: Self-reported measures; ad hoc non-standardized survey.
Friedman 2016 [72] *	To assess the perception of US pediatric specialists of RSV disease risk and determine their clinical practices regarding immunoprophylaxis for high-risk children	USA 2014	Quantitative cross-sectional study. N = 555 MD (203 neonatologists, 138 pediatricians, 58 pediatric pulmonologists, 156 pediatric cardiologists)	A large majority of participants recommended RSV immunoprophylaxis for children at high risk for severe RSV disease. The recommendation and administration of RSV immunoprophylaxis for preterm infants by neonatologists and pediatricians varied with gestational age and the infant’s age at the start of the RSV season. Generally, younger preterm infants were seen as having a greater clinical need for this treatment compared to older preterm infants. Physicians showed mixed agreement with the updates in the 2014 AAP guidance, with most believing that the scientific evidence did not strongly support the changes.	Strengths: Diverse professional background of participants; evaluation of physicians’ attitude towards actual guidelines. Limitations: Sample self-selection bias; self-reported measures; limited validity (no vaccine was offered); ad hoc non-standardized survey.
Haeder 2023 [87]	To query parents about their intention to vaccinate their children against COVID-19, influenza, and RSV in the fall and winter 2023–2024	USA 27–28 September 2023	Quantitative cross-sectional study. N = 616 individuals: parents <8 months (166), expectant mothers (50) and fathers (55), planning on getting pregnant over the next year (345)	71.1% of respondents indicated that they had already or were planning to vaccinate their children against RSV (40.9% against COVID-19 and 63.3% against influenza). Consistent predictors of higher vaccination intention were concerns about the diseases and trust in health institutions. For flu and COVID-19, predictors included previous vaccination against the same disease and the respondent being a man. Most common reasons for hesitancy differed by illness. For RSV, these included concerns about vaccine safety (11.1%), lack of information (10.0%), concerns about vaccine side effects (8.6%), and its necessity (7.7%).	Strengths: Validated instruments; perspective of both parents; multi-center, nationwide study Limitations: Sample self-selection bias; self-reported measures.
Hinderstein 2024 [88]	To understand how parents of healthy newborns would respond to the new recommendation of nirsevimab	USA November 2023–Febuary 2024	Qualitative cross-sectional N = 28 parents of newborns	38% of the 28 parents interviewed planned to give nirsevimab to their newborn, 25% did not, and 38% were uncertain. Key themes: Significant knowledge gaps about RSV prophylaxis, trust in pediatricians, and fear of RSV infection encouraged uptake, and concerns about side effects, timing, and the maternal RSV vaccine were prevalent among those deferring vaccination. Misinformation about nirsevimab, including concerns about its novelty, was common. The Health Beliefs Model was applied to identify ways to positively influence decision-making and address these concerns.	Strengths: Validity (actual vaccination option). Limitations: Sample selection bias; self-reported measures; ad hoc non-standardized data collection procedure.
Knijff 2024 [89]	To compare psychosocial factors of vaccine uptake before (2013) and two years into the COVID-19 pandemic (2022)	Netherlands 2013 and 2022	Quantitative cross-sectional study. N = 2800 parents (2022: 1000 parents (<3.5 years) and 1000 parents (9–14); 2013: 800 parents (<3.5 years)	In both 2022 and 2013, most parents had positive intentions regarding vaccination, with similar levels of trust (around 52%) but slightly lower positive attitudes in 2022 (83.1% vs. 87.0% in 2013). However, in 2022, fewer parents considered vaccination self-evident (57.2% vs. 67.3% in 2013). Negative perceptions increased significantly in 2022, including doubts about vaccine effectiveness, concerns about side effects, preference for natural infection, and reduced trust in the National Immunization Program.	Strengths: Large sample size; perspective of both parents. Limitations: Low response rate at follow-up: sample self-selection bias; limited validity (vaccine was not offered); self-reported measures; ad hoc non-standardized survey.
Kooiman 2019 [79] *	To investigate differences in palivizumab prescription rates between Dutch pediatricians and the role of parent counseling in this practice variation	Netherlands January 2012–July 2014	Quantitative cross-sectional study. N = 208 parents	Overall, 64% received palivizumab, with considerable variability between the three hospitals: 8%, 89%, and 99%. The main differences in counseling in the three practices concerned how the decision was presented (palivizumab is recommended vs. it is a preference-sensitive decision) and the way in which the benefits of immunization were described. In the hospital with the lowest uptake, the decision was presented as a personal choice based on the evaluation of benefits and risks and the benefits were described using the number needed to treat (NNT), specifying that 20 children need to be treated to prevent one hospitalization.	Strengths: Focus on role of counseling on vaccination decision; perspective of both parents Limitations: Sample self-selection bias; self-reported measures.
Langer 2024 [90]	To assess parental knowledge and attitudes towards general childhood and RSV vaccines	Germany Febuary–June 2023	Quantitative cross-sectional study. N = 191 parents (of infants 0–36 months)	3% had never heard of RSV, 39% had only heard of it, and 58% had basic or good knowledge on RSV. 68% would accept RSV immunization, 8% would refuse, and 24% were undecided. Parents who were supportive or undecided about RSV vaccination were less likely to be vaccine-hesitant in general. Parents who refused RSV vaccination were less concerned about the infection compared to vaccine supporters.	Strengths: Perspective of both parents; focus on source of information. Limitations: Sample self-selection bias; self-reported measures; limited validity (vaccine was not offered); ad hoc non-standardized survey.
Langkamp 2001 [83] *	To determine parental factors that are associated with receipt of all monthly doses of palivizumab	USA End of the 1998–1999 RSV season	Quantitative cross-sectional study. N = 385 parents	The overall compliance rate with all recommended doses of palivizumab was 78%. Among the survey respondents, 84% of children had received all doses of palivizumab compared with only 70% of those who did not respond (*p* < 0.001). Factors associated with compliance were parents’ beliefs about its protective effects against RSV (compliant 67% vs. non-compliant 48%), in interaction also with the Medicaid status (parents worrying the most and on Medicaid were more compliant). The main barrier to compliance was transportation issues (compliant 15% vs. non-compliant 35%).	Strengths: Perspective of both parents. Limitations: Low response rate among noncompliants; sample self-selection bias; self-reported measures; ad hoc non-standardized survey.
Lee Mortensen 2022 [91]	To examine parental knowledge about RSV and attitudes regarding RSV immunization	China, France, Germany, Italy, Japan, Spain, UK, US. 18 March–21 May 2021	Quantitative cross-sectional study. N = 5627 parents (<24 months) and expectant parents	74% declared that their children received all recommended childhood vaccines, and 83% intended to continue to do so. 36% had never heard of RSV and 29% had heard the name but knew nothing else (for bronchiolitis, 8% and 21%, respectively). 60% of respondents would likely accept vaccination if it was recommended as part of the immunization program and by the infant’s HCP and information about its efficacy and safety was provided. Most common reason for acceptance: desire to protect children. Most common barrier to acceptance: concerns about potential side effects. Acceptance of the RSV immunization was lower among European and Japanese participants than among Chinese and American participants, for whom HCP recommendation and inclusion in routine immunization programs had a stronger influence on attitude. Parents with more than one child were more hesitant than new parents or parents to be.	Strengths: Large sample size; multi-nation study; perspective of both parents Limitations: Sample self-selection bias; self-reported measures; ad hoc non-standardized survey; funded by pharmaceutical company.
Lee Mortensen 2024 [92]	To gain insights into parental awareness of RSV, their sources of child health information, and attitudes toward infant immunization against RSV in France	France 18 March–21 May 2021	Quantitative cross-sectional study. N = 758 parents (<24 months) or expecting	Focus on data from France provided in Lee Mortensen 2024 [41]. Findings in line with the actual uptake rates of nirsevimab (60–80%) following its introduction in September 2023.	Strengths: Large sample size; perspective of both parents; national study. Limitations: Sample self-selection bias; self-reported measures; ad hoc non-standardized survey; funded by pharmaceutical company.
Lorcy 2020 [73] *	To describe the experience and opinions of HCPs at the end of the first RSV season after the implementation of a new program of prophylaxis	Canada July–September 2017	Qualitative cross-sectional study. N = 20 HCPs	Examination of the feasibility and acceptability issues linked to the implementation of the new RSV prophylaxis program in 2016. Participants identified three main concerns and challenges regarding the new program’s implementation: (a) increased workload, (b) insufficient information on the need and efficacy of palivizumab for healthy full-term newborns, communication problems among stakeholders, and (c) ethical concerns regarding the Inuit population.	Strengths: Focus on actual implementation of an RSV prophylaxis program; healthcare professionals from diverse backgrounds. Limitations: Sample self-selection bias; self-reported measures; ad hoc non-standardized measures.
Moore 2024 [74] *	To measure nurses’ knowledge of RSV and RSV prophylaxis and explore their perceived potential barriers to palivizumab administration to children in an acute hospital setting	Ireland January–Febuary 2021	Quantitative cross-sectional study. N = 144 nurses of children below 1 year in acute hospital setting	96% of respondents recognized the importance of palivizumab for protecting vulnerable infants, but there were gaps in HCPs’ knowledge about monoclonal antibodies for RSV prophylaxis, with 74% mistakenly believing it to be a vaccine. Additionally, 73% correctly understood that palivizumab is administered monthly, but only 36% were aware of the recommended maximum of five doses. The most cited perceived potential barriers to palivizumab administration were uncertainty about which infants are eligible for it, forgetting to check whether a patient is due to receive a dose, parental refusal for treatment because their child is ill, and not knowing the contraindications of palivizumab.	Strengths: Objective measures to evaluate knowledge. Limitations: Sample self-selection bias; ad hoc non-standardized survey.
Pignotti 2006 [84] *	To identify compliance-influencing factors and to suggest strategies for overcoming barriers in a preventive medicine program	Italy November 2000–April 2004	Quantitative cross-sectional study. N = 216 parents of newborns	The overall compliance rate with all recommended doses of palivizumab was 87%. 88% felt that the program was positive and did not consider the monthly schedule to be stressful for their children and 96% would participate in a palivizumab prophylaxis program again if necessary. Compliance was more likely in families of infants born with a lower birth weight or who were younger at the beginning of prophylaxis vs. higher birth weight infants or older children. There was also a statistically significant association between non-native parents and poor compliance (compliance rate 68% vs. 89% in native Italian-speaking parents).	Strengths: Perspective of both parents. Limitations: Self-reported measures; ad hoc non-standardized survey.
Riccò 2022 [75] *	To assess the knowledge, attitudes and practices for RSV in a sample of general practitioners from north-eastern Italy (2021), focusing on the risk perception for infants (age < 8 years) and its potential effectors	Italy 1–15 December 2021	Quantitative cross-sectional study. N = 543 general practitioners	28.7% of respondents had managed at least one RSV case in their practice, 17.8% had diagnosed at least one RSV case and/or recommended the hospitalization, and 5.1% had recommended immunoprophylaxis with monoclonal antibodies. 87.9% characterized RSV as a common disease in infants. 41.4% of GPs knew that monoclonal antibodies could be used only in preventive settings, 35.7% knew that commercially available monoclonal antibodies must be delivered every month during the RSV season, and only 33.8% correctly described the use of monoclonal antibodies in immunoprophylaxis for RSV in pre-term infants. 91.7% expressed willingness to recommend a potential RSV vaccine once it becomes available.	Strengths: Assessment of actual RSV immunoprophylaxis knowlege and counseling. Limitations: Sample self-selection bias; self-reported measures; ad hoc non-standardized survey.
Riccò 2023 [76] *	To assess the knowledge, attitudes, and practices relating to RSV and the preventive use of mAb in a sample of Italian pediatricians.	Italy 7–22 April 2022	Quantitative cross-sectional study. N = 389 pediatricians	Experience with RSV was quite common (41.9% had managed RSV cases in the previous 5 years, 34.4% had diagnosed RSV cases, and 32.6% required a subsequent hospitalization), but only 14.4% had previously required immunoprophylaxis for RSV. Knowledge status was substantially inappropriate (actual estimate 54.0% ± 14.2, potential range 0–100), while the majority of participants acknowledged RSV as a substantial health threat for all infants (84.8%). Attitudes towards RSV prevention were largely positive, with 75% of pediatricians supporting the use of monoclonal antibodies (available the time of the study), and 94% in favor of a potential future vaccine.	Strengths: Assessment of actual knowkedge and counseling for immunoprophylaxis with monoclonal antibodies. Limitations: Sample self-selection bias; self-reported measures; ad hoc non-standardized survey.
Robbins 2002 [85] *	To evaluate the difficulties encountered by parents and barriers that reduce adherence to RSV-IG and palivizumab	USA October 2001–April 2002	Quantitative cross-sectional study. N = 143 parents	Infants receiving RSV immune globuline (RSV-IG) had lower adherence (62%) than those receiving palivizumab (86%). Moreover, in the RSV-IG group infants’ perceived distress and parents’ distress were higher than in the palivizumab group. Discontinued treatment was higher among infants who received RSV-IG (35%) than among those receiving palivizumab (18%)	Strengths: Mixed qualitative and quantitative methods; includes perspective of both parents. Limitations: Sample self-selection bias; self-reported measures; ad hoc non-standardized survey.
Sansone 2024 [93]	To assess parents’ willingness to vaccinate their children with the RSV vaccine and the key predictors of this intention among parents in Italy	Italy April–November 2023	Quantitative cross-sectional study. N = 404 parents <5 years	18.2% of them expressed a high level of concern that their children could be infected with RSV. RSV vaccine administration for children was believed to be very useful to protect them by only 18.2% of the sample. 51.3% of parents expressed their willingness to vaccinate their child. The most frequently mentioned reason to accept vaccination was to protect their children (66.5%), whereas the most common barrier was concern about possible side effects (59.6%).	Strenghts: Multi-nation study; includes perspective of both parents; identification of determinants of source of information. Limitations: Sample self-selection bias; self-reported measures; ad hoc non-standardized survey.
Van Beek 2013 [78] *	To assess the knowledge of healthcare providers in the European Union about RSV infection in Down Syndrome children	Europe Time not specified	Quantitative cross-sectional study. N = 53 caregivers (41 pediatricians, 8 patient associations, 2 nurses, 2 other)	86.7% had knowledge of the increased risk of severe RSV infection in Down Syndrome children. 71.4% responded that it was important to have a statement on the use of RSV prophylaxis in existing guidelines, whereas only a minority of guidelines available at the time of the study did.	Strenghts: Multi-nation study. Limitations: Sample self-selection bias; self-reported measures; ad hoc non-standardized survey.
Wang 2025 [94]	To assess parents’ perceptions of respiratory syncytial virus (RSV) and their attitudes towards the RSV vaccine in China	China 21 August –15 November 2023	Quantitative cross-sectional study. N = 2135 parents (<14 years)	26.0% indicated that they had never heard of RSV (with a higher lack of awareness in parents with a child under 1 year of age, fathers, and those with a non-healthcare-related occupation). 41.2% of participants believed their child was likely to be susceptible to RSV infection. 50.3% of the participants considered RSV infection to be somewhat serious. 70.6% of parents expressed their willingness to vaccinate their child against RSV. Factors associated with higher acceptance: higher level of awareness of RSV, knowledge, and perceptions of susceptibility and severity. Factors associated with lower acceptance: child experiencing side effects after vaccination The most frequent reasons for acceptance were worry about infection and perceived severity. The most common reasons for refusal were concerns about safety and side effect and novelty of vaccine.	Strengths: Large sample size; perspective of both parents Limitations: Sample self-selection bias; self-reported measures; limited validity (vaccine was not offered); ad hoc non-standardized survey.
Weiner 2010 [77] *	To assess the accuracy of information provided by medical practitioners to families regarding the efficacy and limitations of prophylaxis with palivizumab for RSV infection in infants	USA Time not specified	Quantitative cross-sectional study. N = 456 HCPs (neonatologists and pediatricians, neonatal nurse practitioners, neonatal fellows, and newborn intensive care unit nurses)	Almost all clinicians (98%) failed to correctly describe the effect of palivizumab on the severity of the illness, declaring that it reduces the need for ventilation and mortality, while current medical evidence suggests that neither RSV-IVIG nor palivizumab affects the need for mechanical ventilation or mortality.	Strengths: Participants from diverse professional backgrounds. Limitations: Low response rate; sample self-selection bias; self-reported measures; ad hoc non-standardized survey.
Zornoza Moreno 2024 [95]	To provide the first pre-immunization insights into the Spanish parental knowledge about bronchiolitis, RSV, and nirsevimab immunization.	Spain 7–20 September 2023	Quantitative cross-sectional study. N = 3217 parents (<2 years)	Awareness of RSV was moderate, with 46.6% of the respondents knowing about it (higher awareness of bronchiolitis: 95.8%) 64% were concerned about RSV severity, 49% about its frequency in children, and 48% about its contagiousness. Only 11.2% of respondents were aware of nirsevimab. Once informed, the vast majority of participants (97%) would accept nirsevimab immunization for their children. Most frequent reason for acceptance: protection of the infant when most vulnerable (78%). Most frequent reason to refuse: concerns about safety and possible side effects (68%).	Strengths: Large sample size; analysis of source of information. Limitations: Sample self-selection bias; self-reported measures; ad hoc non-standardized survey.

* About palivizumab.

## Data Availability

No new data were created.

## Supplementary Materials

The following supporting information can be downloaded at: https://www.mdpi.com/article/10.3390/vaccines13020159/s1, Search Strategy; Table S1: Articles included in the review (in alphabetical order of first author). Table S2: Percentage of participants who exhibited little or no awareness of RSV (never heard of it or know only the name) and percentage of acceptance of vaccination (uptake and/or intention to vaccinate). Summaries of articles.
