# Supplementary material for: Knowledge and Attitudes Regarding Respiratory Syncytial Virus (RSV) Prevention: A Systematic Review
Source: Vaccines (Basel). 2025 Feb 6;13(2):159. doi: 10.3390/vaccines13020159 (PMC11861692; doi:10.3390/vaccines13020159)
Supplement: Supplementary file 1 [file vaccines-13-00159-s001.zip › vaccines-3359029-supplementary.pdf]

Supplementary material

Search strategy

Searches were performed on the 23rd December 2024

PubMed

("attitude*" OR "intention*" OR "perception*" OR "opinion*" OR "view*" OR "belief*" OR "perspective*" OR "evaluation*" OR "acceptance*" OR "preference*" OR "knowledge" OR "willingness" OR "hesitancy") AND ("respiratory syncytial virus" OR "RSV" OR "human orthopneumovirus" OR "syncytial virus" OR "RSV infection" OR "RSV-associated bronchiolitis" OR "RSV-associated pneumonia" OR "respiratory syncytial virus A" OR "respiratory syncytial virus B") AND ("prevention" OR "prophylaxis" OR "vaccine*" OR "immunization" OR "preventive measures" OR "infection control" OR "protective measures" OR "disease prevention") NOT (systematic review[Publication Type] OR meta-analysis[Publication Type] OR review[Publication Type])

FILTER: FULL-TEXT and ENGLISH

Scopus

TITLE-ABS-KEY ("attitude*" OR "intention*" OR "perception*" OR "opinion*" OR "view*" OR "belief*" OR "perspective*" OR "evaluation*" OR "acceptance*" OR "preference*" OR "knowledge" OR "hesitancy" OR "willingness") AND TITLE-ABS-KEY ("respiratory syncytial virus" OR "RSV" OR "human orthopneumovirus" OR "syncytial virus" OR "RSV infection" OR "RSV-associated bronchiolitis" OR "RSV-associated pneumonia" OR "respiratory syncytial virus A" OR "respiratory syncytial virus B") AND TITLE-ABS-KEY ("prevention" OR "prophylaxis" OR "vaccine*" OR "immunization" OR "preventive measures" OR "infection control" OR "protective measures" OR "disease prevention") AND (LIMIT-TO (DOCTYPE , "ar") OR LIMIT-TO (DOCTYPE , "sh") OR LIMIT-TO (DOCTYPE , "le"))

EBSCO

("attitude*" OR "intention*" OR "perception*" OR "opinion*" OR "view*" OR "belief*" OR "perspective*" OR "evaluation*" OR "acceptance*" OR "preference*" OR "knowledge" OR "willingness" OR "hesitancy") AND ("respiratory syncytial virus" OR "RSV" OR "human orthopneumovirus" OR "syncytial virus" OR "RSV infection" OR "RSV-associated bronchiolitis" OR "RSV-associated pneumonia" OR "respiratory syncytial virus A" OR "respiratory syncytial virus B") AND ("prevention" OR "prophylaxis" OR "vaccine*" OR "immunization" OR "preventive measures" OR "infection control" OR "protective measures" OR "disease prevention")
Limiters: Population Group: Human; Publication Type: Peer Reviewed Journal; English language; Population Group: Human; English Language; Research Article; Exclude MEDLINE records; Language: English; Publication Type: Journal Article;
Expanders: Apply related words; Apply equivalent subjects;
Search modes: Proximity

MedxRxiv

“Attitude” and “RSV”; “Knowledge” and “RSV”

Preprints

“Attitude” and “RSV” ; “Knowledge” and “RSV”

Table S1. Articles included in the review (in alphabetical order of first author).

Ref.	First author	Year	Journal/Repository	Group
[30]	Adhikari	2024	Journal of the Nepal Medical Association	b2) pregnancy and infant
[31]	Al-Jaid	2023	Australian Medical Journal	c) infant only
[32]	Alvaro	2000	Journal of International Medical Research	c) infant only
[33]	Anderson	2009	Patient Preference and Adherence	c) infant only
[34]	Beusterien	2024	Vaccines	b2) pregnancy and infant
[35]	Black	2023	Morbidity and Mortality Weekly Report	a) adult/elderly
[36]	Ciemins	2023	Vaccine	a) adult/elderly
[37]	Congedo	2024	Italian Journal of Pediatrics	c) infant only
[38]	Cubizolles	2023	Vaccine	b1) pregnancy only
[39]	Damatopoulou	2024	Behavioral Medicine	b1) pregnancy only
[40]	de Sentuary	2025	eClinicalMedicine	c) infant only
[41]	Ebersjö	2023	Children	c) infant only
[42]	Erolu	2024	Cardiology in the Young	c) infant only
[43]	Friedman	2016	Clinical pediatrics Journal	c) infant only
[44]	Gagneux-Brunon	2022	Vaccine	b1) pregnancy only
[45]	Giles	2019	Vaccine	b1) pregnancy only
[46]	Haeder	2023	Vaccine	c) infant only
[47]	Haeder	2024	Health Affairs Scholar	a) adult/elderly
[48]	Harteveld	2024	medRxiv	b2) pregnancy and infant
[49]	Hinderstein	2024	Pediatrics	c) infant only
[50]	Holland	2024	Acta Paediatrica	b2) pregnancy and infant
[51]	Hurley	2019	Vaccine	a) adult/elderly
[52]	Kherfan	2023	Vaccines	b1) pregnancy only
[53]	Knijff	2024	Vaccine	c) infant only
[54]	Kooiman	2019	Acta Paediatrica	c) infant only
[55]	La	2024	Human Vaccines & Immunotherapeutics	a) adult/elderly
[56]	Langer	2024	Vaccine	c) infant only
[57]	Langkamp	2001	American Journal of Perinatology	c) infant only
[58]	Lee Mortensen	2024	Archives de Pédiatrie	c) infant only
[59]	Lee Mortensen	2022	Expert Review of Vaccines	c) infant only
[60]	Limaye	2024	Health Policy and Planning	b1) pregnancy only
[61]	Limaye	2023	Human Vaccines & Immunotherapeutics	b1) pregnancy only
[62]	Limaye	2023	Journal of the Pediatric Infectious Diseases Society	b1) pregnancy only

[63]	Limaye	2024	BMC pregnancy and childbirth	b1) pregnancy only
[64]	Lorcy	2020	International Journal of Circumpolar Health	c) infant only
[65]	Maculaitis	2024	Human Vaccines & Immunotherapeutics	b2) pregnancy and infant
[66]	McCormack	2024	Immunity, Inflammation and Disease	b1) pregnancy only
[67]	Miraglia del Giudice	2023	Vaccines	b2) pregnancy and infant
[68]	Moore	2024	Nursing children and young people	c) infant only
[69]	Motta	2025	Public Health	a) adults/elderly
[70]	Nyawanda	2023	Vaccines	b1) pregnancy only
[71]	Papagiannis	2024	Healthcare	a) adults/elderly
[72]	Paulson	2024	medRxiv	b2) pregnancy and infant
[73]	Pignotti	2006	The Journal of Maternal-Fetal & Neonatal Medicine	c) infant only
[74]	Ponticelli	2024	Infectious Disease Reports	a) adult/elderly
[75]	Riccò	2023	Pediatric Reports	c) infant only
[76]	Riccò	2022	Pediatric Reports	c) infant only
[77]	Robbins	2002	Ambulatory Pediatrics	c) infant only
[78]	Sallam	2024	Preprints	b1) pregnancy only
[79]	Sansone	2024	Vaccines	c) infant only
[80]	Saper	2024	Pediatrics	b1) pregnancy only
[81]	Singh	2024	Maternal and Child Health Journal	b1) pregnancy only
[82]	Strózik	2024	Preprints	b1) pregnancy only
[83]	Tucker	2024	O&G Open	b2) pregnancy and infant
[84]	van Beek	2013	Journal of Immunology Research	c) infant only
[85]	Vascimini	2024	Journal of Pharmacy Technology	a) adult/elderly
[86]	Wang	2024	Human Vaccines & Immunotherapeutics	a) adult/elderly
[87]	Wang	2025	Vaccine	c) infant only
[88]	Weiner	2010	American Journal of Perinatology	c) infant only
[89]	Wilcox	2019	The Pediatric infectious disease journal	b1) pregnancy only
[90]	Zornoza Moreno	2024	Human Vaccines & Immunotherapeutics	c) infant only

Table S2. Percentage of participants who exhibited little or no awareness of RSV (never heard of it or know only the name) and percentage of acceptance of vaccination (uptake and/or intention to vaccinate).

Article	Little or no awareness of RSV	Acceptance of vaccination
**Adult vaccination**		
Wang 2024 [86]	65%	68%
La 2024 [55]	65%	-
Black 2023 [35]	-	31%
Header 2024 [47]	-	51%
Motta 2025 [69]	-	46%
Vascimini 2024 [85]	-	63%
**Maternal vaccination**		
Strózik 2024 [82]	2%	16%
Holland2024* [50]	10%[a]<br>21%[b]	80%[a]<br>87%[b]
Saper2023 [80]	20%	54%
Paulson 2024* [72]	30%	88%
Sallam 2024 [78]	46%	78%
Harteveld 2024* [48]	54%	67%
Miraglia del Giudice 2023* [67]	65%	46%
Damatopoulou 2024 [39]	76%	18%[c]
		45%
McCormack 2024 [66]	76%	49%
Giles 2019 [45]	83%	77%
Wilcox 2019 [89]	88%	29%[c]
		75%
Adhikari 2024* [30]	98%	42%
**Infant vaccination**		
Wang 2025 [87]	26%	71%
Sansone 2024 [79]	42%	51%
Langer 2024 [56]	42%	68%
Al-Jaid 2023 [31]	49%	45%
Haeder 2023 [46]	-	71%
Zornoza Moreno 2024 [90]	53%	97%
Lee Mortensen 2022 [59]	65%	60%
Hinderstein 2024 [49]	-	38%
Knijff 2024 [53]	-	65%[d]
	-	73%[e]
de Sentuary 2025 [40]	-	92%

[a] current parents; [b] future parents; [c] if RSV was in a clinical trial; [d] 2013 data; [e] 2022 data; * maternal and infant articles

Summaries of articles

a) Knowledge and attitudes towards RSV vaccines for elderly and adults at risk

In a brief commentary, **Ciemins** and colleagues examined knowledge of RSV and attitudes toward a vaccine among a small group of American clinicians [36]. They gathered data from a questionnaire (n = 30) and roundtables or interviews (n = 44). The results revealed a moderate level of knowledge about RSV, while qualitative insights indicated a strong desire for more information to better educate patients on preventive measures, including vaccination. The authors emphasized that official recommendations from trusted institutions are crucial for shaping local guidance. Clinicians' perspective about their patients' views about vaccines was twofold, imagining the persistence of hesitancy towards vaccines that emerged during the pandemic, while also anticipating that the uptake of the RSV vaccine might mirror that of the influenza vaccine.

The other studies on healthcare professionals used surveys. One study was conducted in 2017 in the USA by **Hurley** and colleagues and recruited over 300 physicians involved in primary care who reported having treated patients with RSV in the previous year [51]. Despite experience in caring for patients with RSV, most physicians (86%) would like more information about the burden of RSV in adult patients. At the same time, the majority (74%) stated that influenza is generally more severe than RSV among patients aged over 50 years and that they rarely consider RSV as a potential pathogen in patients 50 years or older with a respiratory disease (57%). As for a potential vaccine, participants anticipated that the more common barriers for patients would be economical, namely lack of insurance cover and lack of adequate reimbursement.

To inform strategies to enhance vaccine acceptance and implementation, in response to the potential introduction of new RSV vaccination guidelines by the Greek National Vaccines Committee, **Papagiannis** and colleagues surveyed over 200 Greek HCPs between October and December 2023 [71]. About 65% of HCPs indicated that they felt adequately informed about the vaccine and about 70% correctly identified the vaccine's current availability, with 62% aware of the current recommendation for RSV vaccination in pregnant women. Nonetheless, about half (48%) incorrectly indicated that vaccination was available for infants up to 6 months, children, and adolescents and about one-third incorrectly indicated that passive immunity could serve as immune prophylaxis against RSV.

Ponticelli and colleagues focused on healthcare professionals in the field of cardiology (n = 154, 99 nurses and 55 physicians) to assess their knowledge about and attitude towards RSV and RSV vaccines, as well as their intention to recommend vaccination to their patients in Italy in November 2023 [74]. The findings indicated that knowledge about RSV was somewhat limited, with over 90% of participants expressing a desire for more information on vaccination for older adults and chronic patients, as well as details about the RSV vaccine itself. Healthcare professionals with higher levels of education and a more positive attitude towards vaccine safety were more likely to have positive attitudes towards RSV vaccination. Additionally, being a physician and being aware of new RSV vaccines that had received market authorization were associated with a greater willingness to recommend or suggest vaccination to patients. However, concerningly, about three-quarters of the cardiac healthcare professionals believed that patients would be reluctant to accept an additional vaccine due to safety concerns, and roughly half felt that patients were being offered too many vaccinations.

Wang and colleagues investigated attitudes towards RSV and its vaccination in a convenience sample of the general population (n = 2133) in China during August and September 2023 [86]. The sample included adults of all ages, but only a small fraction (n = 115, 5.4%) was above 50 years old. Overall, awareness and knowledge about RSV were low, with approximately 25% of participants having never heard of it and 40% familiar with the term but unaware of its significance and its consequences. More than half of the participants (56%) had a low individual risk perception while three-quarters (77%) perceived RSV infection as having low severity. Surprisingly only one-third of participants (32%) expressed reluctance to get vaccinated, citing concerns about the vaccine's safety and its novelty as primary reasons for their hesitation. In contrast, worries about the risks associated with RSV infection and a desire to protect those around them were the most common reasons for accepting the vaccine. Indeed, individuals living with children under the age of 6 or adults over 60 years older were more likely to be open to receiving the RSV vaccine. However, those aged over 50 years were less inclined to get vaccinated themselves and exhibited low levels of perceived susceptibility and severity regarding the disease.

Age is a significant risk factor for severe RSV, leading most guidelines to recommend vaccination for individuals above a certain age (typically 60, 65 or 75), as well as for all adults with risk factors who are also at increased risk of severe consequences from RSV infection. Before the introduction of RSV vaccines, **La** and

colleagues considered both people at risk due to age as well as those with one or more chronic condition in the USA in May-June 2022 [55]. Specifically, they surveyed a total of 827 people divided in 4 groups: adults aged 60–89 years (n = 224), adults aged 18–59 years with a chronic cardiovascular condition (n = 200), with a chronic pulmonary condition (n = 347), and with diabetes mellitus (n = 308). The authors assessed participants' knowledge, attitudes and perceptions of RSV illness but they did not assess attitudes towards RSV vaccination. Despite their increased risk and the fact that about three-quarters considered themselves knowledgeable about respiratory infections, only 43% of participants had ever heard of RSV and only 35% of those aware of RSV reported being knowledgeable about it. Indeed, knowledge was rather poor, with issues also around the bacterial vs. viral nature of respiratory infections, RSV seasonality, and common RSV symptoms. Among RSV-aware adults, one-third declared to be (very) worried about RSV, although this concern rarely led them to consider RSV as a potential cause of their cold/flu-like symptoms.

In the USA, two RSV vaccines were introduced in June 2023, indicated for people aged 60 and older and recommended by the CDC's Advisory Commitee on Immunization Practices. At the end of September 2023, **Haeder** evaluated RSV vaccination status and intent to vaccinate in a large, nationally representative sample, which included 1,345 participants aged 60 and older [47]. Approximately half of the sample indicated they planned to get vaccinated in the upcoming season (42%) or reported having already received the vaccine (9%). Consistent with existing literature, women were less likely to be vaccinated. Among those who had not yet been vaccinated, the intention to receive the vaccine was influenced by concerns about the disease and perceived individual risk, as well as trust in health institutions and the safety of vaccines. Conversely, the most common reason for refusing vaccination was a lack of perceived necessity (25%), followed by insufficient information (24%), concerns about side effects (22%), and safety concerns (13%).

Likewise, **Vascimini** and colleagues recruited 518 participants between September 2023 and March 2024 in community pharmacies in the USA [85]. Most participants had not received education about RSV by their healthcare professionals (78%), nonetheless, between 55 and 70% were knowledgeable about RSV transmission, ways to protect and lack of immunity from previous infections. Once informed, a majority of this sample (63%) decided to receive the vaccine after filling the survey.

Between October and November 2023, **Motta** and colleagues recruited a representative sample of the US adult population and considered answers of the 362 people over 60 years old for their study [69]. Only a minority of participants had already received (14%) or booked vaccination (5%), with an additional 27% planning to do so in the future, whereas over half of participants planned to forgo vaccination (53%). Vaccination behavior was predicted by previous vaccination behavior for flu and COVID and by perceived efficacy and safety of the RSV vaccine.

Similarly, **Black** and colleagues collected data between the end of September and early December 2023 on a very large sample (N=62816 for RSV) in the USA [35]. Participants were asked about their vaccination status for COVID-19, influenza, and RSV. Those who were unvaccinated were also queried about their intentions to get vaccinated. Over the study period, the proportion of participants who were unvaccinated but stated they would definitely get vaccinated reduced significantly, particularly for influenza (from 33.2% to 9.4%) and COVID-19 (from 28.2% to 14.1%). A similar trend was observed for RSV, which decreased from 20.9% to 14.1%. By the end of the study, among participants aged 60 years and older, 17.0% had been vaccinated against RSV, while 14.1% indicated they would definitely get vaccinated. Additionally, 38.7% either intended to vaccinate or were undecided, and 30.2% stated they probably would not vaccinate. In comparison, among those aged 65 years and older, 69.3% were vaccinated against influenza, with 8.2% intending to get vaccinated, and 37.4% had been vaccinated against COVID-19, while 15.7% intended to do so.

b1) Attitudes towards RSV maternal vaccines

Among the studies targeting healthcare professionals, two were conducted in Kenya utilizing in-depth interviews only [62] or together with a questionnaire [70]. The other two studies focused on participation in clinical trials for RSV vaccines and were conducted in France and in the UK [42,55].

In a mixed method study, **Nyawanda** and colleagues examined current knowledge, attitudes, and perceptions around RSV disease and prevention among the HCPs in Kenya [70]. Between September and October 2021, 106 healthcare workers (94 front line HCPs and 12 health management officers) participated in in-depth interviews and completed a questionnaire. Awareness of RSV was rather low, with only 39% having heard about RSV. Almost no participant (2%) was aware of RSV prevention products either available or in development. Despite this, almost all participants (93%) who were aware of RSV felt that pregnant

women should be vaccinated against RSV, most recommending a single-dose vaccine schedule (59%) mainly for maximal adherence and compliance.

Between August and September 2022, **Limaye** and colleagues interviewed 16 providers to inform future maternal RSV vaccine rollout based on insights from the COVID-19 vaccine experience [62]. They emphasized the critical importance of community sensitization as the key lesson learned from the COVID-19 vaccine rollout. They identified three essential elements: a) communication to ensure that the community is aware of the risks of RSV harms and the benefits of RSV maternal vaccines, including wide dissemination of clear and comprehensive information, particularly regarding the safety of vaccines during pregnancy; b) mobilization of healthcare providers and community leaders for building trust within communities and integrating vaccines into routine healthcare schedules; and c) education of pregnant women focusing on the safety of vaccines for both themselves and their unborn children, the duration of protection, and the appropriate vaccine dosing.

As the pandemic highlighted, pregnant women have historically been excluded from vaccine clinical trials, despite their inclusion being crucial during health crises. **Gagneux-Brunon** and colleagues surveyed nearly 400 midwives from September to November 2020 to investigate the willingness of midwives to encourage pregnant individuals to participate in a hypothetical RSV vaccine clinical trial in France [44]. Only 28% of midwives expressed a willingness to promote such participation, with the sole predictive factor being their experience with vaccine education.

In contrast, in a study by **Wilcox** and colleagues, involving around 200 healthcare professionals and over 300 pregnant women between July 2017 and January 2018 in the UK, a greater proportion of midwives (68%) supported clinical trials, though their backing was lower than that of obstetricians (92%) [89]. Additionally, midwives were less likely to endorse routine RSV vaccination (79% compared to 89% for obstetricians). Notably, a significant percentage of midwives reported having little to no knowledge about RSV (66% vs. 14% for obstetricians). In the same study, while most pregnant women (88%) were either unaware of RSV or had minimal knowledge about it, a relatively high percentage (29%) indicated they would participate in an RSV vaccination trial. Furthermore, 75% of pregnant women expressed willingness to receive the vaccine if it were part of the routine immunizations recommended during pregnancy.

Among the studies focusing on pregnant women, four employed qualitative methods [60,61,78,81]. Two of these studies were conducted in Kenya by the group lead by **Limaye**. Both used semi-structured interviews between July and September 2022, with a small sample consisting of pregnant women (n = 6) and lactating women (n = 18). One of them explored awareness of RSV, its causes, risk perceptions, and key questions regarding future maternal RSV vaccines [60]. The other study also included community members (n = 10) and examined the decision-making process surrounding the RSV maternal vaccine. It emphasized that the pregnant woman was viewed as the primary decision-maker and that healthcare professionals were recognized as crucial sources of information and influential figures in the decision-making process [78]. The same research group also explored the perspectives of 20 policymakers in Kenya [63]. In-depth interviews revealed that the development of policy and the introduction of a new vaccine must be grounded in substantial evidence and coordinated among all stakeholders. Additionally, the sustainability of the new policy is crucial, and educating communities is especially important, given that the target group—pregnant women—is particularly sensitive.

The other qualitative study was conducted in Jordan between January and February 2024 and utilized face-to-face semi-structured interviews with over 400 pregnant women [78]. The aim was to assess their willingness to receive the RSV vaccine and identify associated determinants using a previously validated scale [52]. **Sallam** and colleagues found that while only about half of the participants (54%) had heard of RSV prior to the study, a significant majority (78%) expressed willingness to get vaccinated if the vaccine was safe, effective, and free. Consistent with existing literature, maternal attitudes toward RSV vaccination were influenced by several factors, including demographic variables, vaccination history, trust in health institutions, concerns about vaccine safety, and fears regarding the potential consequences of the disease in infants.

The remaining studies employed quantitative methods. **Limaye** and colleagues, building on their qualitative work and applying the behavioral and social drivers of vaccination framework (BeSD) [91,92] assessed a total of 400 participants (25% pregnant individuals, 75% lactating women) in the fall 2022 [61]. The findings revealed that a substantial majority of participants held higher perceptions of RSV prevalence (87%) and risk (54%). Additionally, they reported strong supportive social norms (80%), high self-efficacy (87%), and low perceived barriers to vaccination (83%). Confidence in vaccine safety was notably high at 97%, with 89% expressing trust in vaccine effectiveness. Interestingly, participants experiencing their first

pregnancy or child were more hesitant about vaccination compared to those with prior experience. Furthermore, social norms also played a role in predicting vaccine hesitancy among lactating women.

McCormack and colleagues conducted their research in Ireland between December 2018 and April 2019, recruiting a sample of 528 pregnant women [66]. Consistent with other studies, awareness of RSV was notably low, with 76% of participants having never heard of the virus. However, nearly half (49%) expressed willingness to receive the vaccine if it were included in the national immunization plan. The primary predictor of acceptance was the perceived protection the vaccine would offer for the infant.

In March 2023, **Saper** and colleagues conducted a survey of over 1,500 respondents in the USA to assess interest in RSV vaccination during pregnancy. The sample included individuals who were either currently pregnant (52%) or planning to become pregnant within the year (48%) [80]. Unlike other studies that reported low awareness of RSV, only a minority of respondents (20%) in this sample had never heard of the virus. However, the willingness to receive the RSV maternal vaccine (54%) was consistent with findings from other studies. The perceived severity of RSV for infants emerged as the primary predictor of vaccine acceptance, along with the presence of other children in the household.

Between April and December 2023, **Damatopoulou** and colleagues recruited 335 pregnant women in Greece to gauge their interest in RSV vaccination during pregnancy if it was a clinical trial and if it was part of the national immunization plan [39]. The majority of participants were unaware of RSV (75.5%). A minority (18.3%) would consent to be vaccinated against RSV during pregnancy if it was a clinical trial, whereas if it was recommended almost half would accept it (44.8%).

Strózik and colleagues assessed the uptake of RSV vaccine during pregnancy in a sample of 668 recently pregnant or pregnant women in Poland [82]. The study was conducted between late July and early August 2024. Overall, 16% of participants reported having been vaccinated against RSV during pregnancy. Women in a medical profession were more likely to have received the vaccine (25%) than those in other professions (13%). The likelihood to have been vaccinated was also higher among participants who had been informed by their physicians about the possibility to vaccinate (25% vs. 10%) and among those who had received other vaccinations during pregnancy (23% vs. 2%).

The other studies explored attitudes toward various maternal vaccinations in addition to RSV, specifically focusing on Group B streptococcus (GBS) [45], seasonal influenza, COVID-19, and pertussis [38]. **Giles** and colleagues surveyed nearly 500 pregnant women in Australia between November 2017 and June 2018 [45]. Most participants were unfamiliar with RSV (83%) or GBS (63%), but after receiving information, a significant proportion expressed a strong intention to vaccinate against RSV (77%) and GBS (74%). Previous acceptance of maternal vaccines for influenza and pertussis was linked to a higher intention to vaccinate. Consistent with earlier findings on maternal COVID-19 vaccination [94], women showed greater interest in the benefits of vaccination for their babies rather than for themselves, although their primary concern remained the safety of the vaccine for both themselves and their infants.

Cubizolles and colleagues recruited over 300 pregnant women in France between February and May 2023 to assess their knowledge and attitudes toward vaccination against influenza, COVID-19, and RSV during pregnancy [38]. They employed the 5C model of vaccine hesitancy (comprising confidence, complacency, constraints, calculation, and collective responsibility) [93] to explore factors influencing intentions to get vaccinated. Intentions to vaccinate were similar across the four diseases, with slightly higher rates for influenza (44%), followed by RSV (39%), COVID-19 (37%), and pertussis (36%). In comparing these estimates with those obtained in other studies it is important to note that France is among the European countries with the highest levels of vaccine hesitancy. In line with existing literature [94,95], confidence in vaccines emerged as the most significant predictor of vaccination intentions according to the 5C model [93]. This confidence was linked to intentions to vaccinate against influenza, RSV, and COVID-19 during pregnancy. Concerns about vaccine safety, particularly regarding potential risks to the baby, were identified as the primary barriers to maternal vaccination.

Finally, one article reported on the development and validation of an RSV vaccine acceptance scale that was conducted in Jordan with a sample of 315 young females recruited in July 2023 [52]. The 21-item survey instrument was named "ABCDEF," reflecting the six sub-scales identified through principal component analysis: Advice, Burden, Conspiracy, Dangers, Efficiency, and Fear.

<u>b2) Attitudes towards RSV maternal vaccines and towards RSV immunization for infants</u>

Adhikari and colleagues utilized semi-structured interviews to collect data in Nepal, between October and November 2023 [30]. The authors recruited over 300 pregnant women to examine their knowledge of RSV and their willingness to accept vaccines during pregnancy, while also considering their practices and

knowledge regarding other vaccinations [30]. In this study, only 2% of participants were aware of RSV before the study, although many had heard of bronchiolitis without understanding what it entails (83%). However, after receiving information, 42% indicated they would definitely be willing to receive the RSV vaccine during pregnancy. The majority (72%) preferred a maternal vaccine over infant immunization (28%).

In a very interesting work, **Beusterien** and colleagues used a discrete choice experiment (DCE) to gauge the relevance that participants gave to different attributes of a hypothetical RSV preventive option [34]. The sample included almost 1,000 pregnant people and over 300 HCPs who cared for pregnant people and/or infants. Data were collected between October and November 2022 in the USA. Participants were presented with 12 choices between three options: two derived from the combinations of different levels of 5 attributes of potential RSV prevention and the third was always the "no preventive product" option. One of the attributes examined was the preventive type: whether prevention was provided via a maternal vaccine administered during pregnancy or a monoclonal antibody administered to infants. The other attributes considered were: effectiveness (50%, 63%, 77%, 90% protection against serious illness requiring hospitalization), injection recipient/timing (during pregnancy, within 14 days after birth, 2-4 months after birth), type of medical visit required to receive the injection (routine or extra check-up appointment), and duration of protection during RSV season (1, 3, 4.5 or 6 months). Moreover, in order to explore the impact of additional attributes not included in the DCE, a best–worst scaling (BWS) exercise was used. Specifically, participants were presented with a list of attributes and asked to pick the two that would make them more (and least) likely to accept the preventive option that included it. To note that the attributes considered by Beusterien and colleagues did not include safety and potential side effects. The findings showed that most of the time a preventive option is preferred to a no preventive option by both pregnant women (89%) and HCPs (96%), no matter what the attributes of the preventive option are. The attributes that had the strongest impact on participants' choice in the DCE were the effectiveness in preventing severe RSV illness (relative importance [RI] in pregnant women 48%, in HCPs 42%) and the duration of protection during the RSV season (RI in pregnant women 28%, in HCPs 35%). The more favorable the level of an attribute, the stronger was the preference for it. The other three attributes used in the DCE had a smaller impact on the choice, for both pregnant women and HCPs. Not surprisingly routine check-up was preferred over an extra appointment to receive the injection (RI in both samples 5%). Interestingly, vaccine was preferred over monoclonal antibody as the preventive type (RI in pregnant women 6%, in HCPs 7%) and pregnant person was preferred over infant as the injection recipient (RI in pregnant women 14%, in HCPs 11%). From the BWS exercise, the 5 top ranked attributes by both samples concerned all the protection of the infant (including the length of the protection, the protection of both pre-term and full-term infants, and the protection starting at birth or at the beginning of the RSV season).

Maculaitis and colleagues performed a secondary analysis of the data collected on pregnant women by Beusterien and colleagues [34] using latent class analysis (LCA) to identify subgroups of pregnant people with different preferences for RSV preventive options [65]. This analysis identified three preference subgroups, whose choice was mainly driven by: effectiveness (51%), duration of protection during the RSV season (39%), and preference for no-preventive option (9%). Interestingly, while women in the first two subgroups preferred a maternal vaccine over monoclonal antibody, the opposite was true for women who often preferred the no-preventive option, who were more favorable to monoclonal antibody than a maternal vaccine.

The awareness of RSV and acceptance of maternal and infant prevention strategies was assessed in almost 500 Italian pregnant women between April and June 2023 [67]. Awareness of RSV was low, with only a third of pregnant women having heard about the RSV infection, and more than two-thirds of them expressing the need to receive additional information about prevention strategies against RSV. After being provided information, risk perception remained low, with only 20% of pregnant women very concerned about RSV infection. Attitudes toward prevention strategies were rather unenthusiastic, with a minority considering them very useful to protect the newborn (16% for maternal vaccination and 22% for infant immunization). In this sample, willingness to undergo maternal vaccination was lower (46%) than willingness to immunize the newborn (61%). In both cases protecting the child was the main reason to accept (71% for maternal vaccine and 76% for infant immunization), while the main reason to refuse was being concerned about side effects (51% for maternal vaccine and 42% for infant immunization).

Between February and April 2024 **Harteveld** and colleagues recruited over 1000 pregnant parents in the Netherland to assess their perception and willingness to accept maternal vaccination or neonatal immunization against RSV [48]. The sample held positive attitudes towards vaccination in general, with 91% being or intending to be vaccinated against Tdap during pregnancy and 94% intending to vaccinate their

newborn. RSV was quite known, with 46% of participants knowing it well and 41% having heard of it. RSV prevention was quite accepted, with the majority of participants willing to accept for sure a maternal vaccine (67%), infant immunization (64%) with a vast overlap (56% willing to accept both for sure) and an additional 31% leaning towards acceptance of both. When asked directly the preference for the type of prevention, a large majority of pregnant women (75%) preferred maternal vaccination alone (75%) or combined with infant immunization (8%) and almost none (3%) preferred infant immunization only. Similar results were obtained from their partners, with 72% preferring maternal vaccination. In line with other findings, decision-making about RSV prevention was guided by the desire to protect the child (87%), followed by concerns for the severity of RSV infection (75%), whereas main reasons for refusal or hesitancy about it were lack of RSV knowledge (43-56%) and fear for the unborn child's safety (23-34%).

One of the few studies that collected data on real uptake of RSV vaccination was conducted in the USA between September 2023 and January 2024, during the first season in which it was offered to pregnant individuals [83]. In this study, 206 participants who qualified to be offered the vaccine were identified and those who refused it were offered to participate in a phone interview. A little more than half of the pregnant women were offered the vaccine (n = 111, 54%). Among them, the acceptance rate was 56% (n = 62). Among participants who refused RSV vaccination during pregnancy (n = 49, 44%), 35 (71%) participated in the interview. Although the sample was small, it confirmed the results of other studies in a real setting: the main reason to decline vaccination was distrust of new vaccines (80%), followed by fear of side effects on the fetus (46%) also in the long-term (23%), that was not limited to this vaccination in pregnancy (17%). Indeed, a greater proportion of women who declined RSV vaccination had also declined other maternal vaccines compared to those who accepted it (Tdap: 35% vs. 2%; Flu: 74% vs. 49%; COVID-19: 84% vs. 18%). While almost all decliners (94%) reported that their clinician adequately discussed the vaccine, about one-third (34%) felt that they had not enough time to decide. Despite declining vaccination during pregnancy, the majority of interviewees would accept newborn RSV antibody (63%) and would likely accept the vaccine in a future pregnancy (66%).

While the previously mentioned studies on attitudes towards RSV prevention during pregnancy and for infants included pregnant participants only, the last two studies included also parents. Specifically, between July and August 2022, **Holland** and colleagues assessed parental awareness of RSV and the level of acceptance of future RSV prevention strategies in Australia [50]. The sample included current parents of children aged ≤5 (n = 1931) and pregnant/planning parents (n = 464, of whom 403 were also current parents and 61 only future parents). Participants' awareness of a series of respiratory illnesses and pathogens was explored. Most participants were aware of most respiratory illnesses (92-95% for influenza, whooping cough, pneumonia, COVID-19, common cold, and asthma), whereas the awareness of pathogens was less well established. Awareness of bronchiolitis was common (92% current parents, 84% future parents) as was awareness of RSV (90% current parents, 79% future parents), although the associated conditions were less well known, especially among future parents (e.g., awareness that pneumonia is associated with RSV was 64% in current parents and 50% in future parents). In contrast with other studies that assessed acceptance of prevention strategies after providing information about them, in this study acceptance was examined both before and after providing information to those who were not aware of RSV (n = 97 current parents and n = 10 future parents). Interestingly, even before receiving the information the majority already accepted a preventive strategy. Overall, the majority of participants would accept maternal vaccination (80% among all pregnant/planning parents, 87% among first-time future parents). Similarly, the majority of participants would accept infant immunization (81% current parents, 93% future parents). The small subsample (n = 57) of future parents who were favorable to both immunization strategies showed a clear preference for the maternal vaccine, either in combination with infant immunization (49%) or alone (39%), whereas infant immunization only was rarely preferred (4%).

Finally, **Paulson** and colleagues explored views regarding the different methods of protecting infants against RSV in a sample of 1620 individuals that were parents of children up to 2 years and/or currently pregnant [72]. Data were collected in the UK between August and September 2023. Similarly to other studies RSV was less commonly known than other viruses and less heard of than bronchiolitis, with 11% having never heard of it and 19% having heard of it but not knowing what it is. Despite this gap in awareness, attitudes towards prevention were very positive, with the majority of participants willing to accept a vaccine in pregnancy (88%) and infant monoclonal antibody (78%). Offering protection for newborns at birth in hospitals did not change acceptability (79%). Not only the acceptance of a maternal vaccine was higher than the acceptance of infant monoclonal antibody, but when asked directly to express a preference, most participants (83%) would prefer the maternal vaccine during pregnancy over an infant antibody. Similarly to

other studies, the desire to protect the baby was the most common reason in favor of RSV prevention, whereas safety concerns were the most common reasons against it.

c) Attitudes towards RSV immunization for infants

Among the articles reporting on HCPs samples, three studies investigated knowledge, attitudes, and practices regarding RSV and its prevention among Italian pediatricians [32,37,75]. One of the oldest studies surveyed 344 Italian pediatricians working in pediatrics, neonatology or intensive care units in 1999 and found that only half of them were aware of the possibility to administer palivizumab [32].

More recently, in April 2022, **Riccò** and colleagues surveyed nearly 400 pediatricians, thoroughly assessing knowledge (through 19 true or false and 6 multiple choice questions based on existing literature), risk perception (gauging perceived probability and perceived severity of RSV separately for infants, adults, and elderly), attitudes towards monoclonal antibodies, and practices (experience in the past 5 years linked to RSV diagnosis, management, hospitalization, and immunoprophylaxis) [75]. The results indicated that experience with RSV was not uncommon: 42% of participants reported managing one or more RSV cases, 34% participated in diagnosing it, and 33% hospitalized patients with RSV. However, only a small minority (14%) prescribed immunization with palivizumab. Overall knowledge was found to be lacking, particularly in several critical areas. Many pediatricians were unaware that approximately 60% of pediatric low respiratory tract infections are associated with RSV, mistakenly believed that the majority of hospitalizations occur primarily in at-risk children (preterm infants and those with chronic respiratory conditions or cardiac malformations), and underestimated the global burden of RSV-related deaths in children under one year old. Despite these knowledge gaps, attitudes towards RSV prevention were largely positive, with 75% of pediatricians supporting the use of monoclonal antibodies (available the time of the study), and 94% in favor of a potential future vaccine.

One year later, from February to May 2023, **Congedo** and colleagues recruited approximately 500 pediatricians through the Italian Society of Paediatrics [37]. Similar to Riccò's findings, while 98% of pediatricians reported managing bronchiolitis cases, only 44% prescribed monoclonal antibodies. Moreover, knowledge among the participants was relatively poor, with 36% unaware that passive immunization to prevent RSV is available for infants under two years, and 25% mistakenly believing that active immunization was currently available. The study also assessed attitudes toward RSV prevention by measuring support for: a) the use of a vaccine against RSV if it were available, safe, and cost-effective; b) the administration of monoclonal antibodies to all newborns and children during their first RSV season, given similar conditions; and c) the implementation of an RSV prevention strategy for all newborns and children to help prevent complications such as bronchospasm and asthma. Reflecting Riccò's findings, the vast majority of pediatricians (92%) expressed support for an RSV vaccine, slightly fewer (82%) favored the use of monoclonal antibodies in infants during their first RSV season, and the vast majority (92%) supported a prevention strategy for all infants to help prevent complications. Positive attitudes towards RSV prevention were associated with greater knowledge about monoclonal antibodies and RSV, as well as being female.

The other article examining Italian healthcare professionals' attitudes towards RSV prevention focused on 157 general practitioners (GPs) recruited in Italy in December 2021 [76]. Approximately one-third of GPs reported having managed at least one case of RSV in their practice; however, only 5% had recommended immunoprophylaxis with monoclonal antibodies. Knowledge levels among the participants were generally low, with significant gaps in RSV epidemiology and prevention. For instance, only 22% correctly identified that most RSV-related deaths occur in the elderly, while over 60% believed that RSV infection was confined to infants and children. Additionally, 20-30% thought that most hospitalizations were among at-risk children. Despite these knowledge gaps, an overwhelming 92% of participants expressed willingness to recommend a potential RSV vaccine once it becomes available.

During the fall 2016 in Nunavik (Quebec, Canada) eligibility for palivizumab was expanded to include healthy full-term newborns because of their high RSV hospitalization rates. **Lorcy** and colleagues conducted semi-structured interviews with 20 HCWs involved in the implementation of this change, in order to improve the new program [64]. The study identified notable feasibility and acceptability issues, highlighting that the program was resource intensive. Key challenges included increased workload, insufficient information on the need and efficacy of palivizumab for healthy full-term newborns, communication problems among stakeholders, and ethical concerns regarding the Inuit population.

The study by **Friedman** and colleagues assessed the perception of US pediatric specialists of RSV disease risk and its prevention and explored changes in physician use of RSV immunoprophylaxis following

a change in guidance that was issued in 2014 by the American Academy of Pediatrics (AAP) [43]. Specifically, they recruited a total of 555 pediatricians in four specialties (neonatologists, pediatricians, pediatric pulmonologists, and pediatric cardiologists) and assessed their perspectives before and after the change in guidance. A large majority of participants recommend RSV immunoprophylaxis for children at high risk for severe RSV disease, The recommendation and administration of RSV immunoprophylaxis for preterm infants by neonatologists and pediatricians varied with gestational age and the infant's age at the start of the RSV season. Generally, younger preterm infants were seen as having a greater clinical need for this treatment compared to older preterm infants. Physicians showed mixed agreement with the updates in the 2014 AAP guidance, with most believing that the scientific evidence did not strongly support the changes.

Weiner and colleagues examined the accuracy of information provided by medical practitioners to families regarding the efficacy and limitations of prophylaxis with palivizumab in infants in the USA [88]. To do so, they surveyed 456 healthcare professionals with experience in palivizumab prescription, including neonatologists and pediatricians, neonatal nurse practitioners, neonatal fellows and newborn intensive care unit nurses. The results suggest that almost all clinicians (98%) failed to correctly describe the effect of palivizumab on the severity of the illness, declaring that it reduces the need for ventilation and mortality.

Similarly, but from the patients' perspective, **Kooiman** and colleagues assessed the contribution of parent counseling about RSV immunization on the difference in palivizumab prescription rates between Dutch pediatricians [54]. They performed a retrospective chart review of all preterm infants born between January 2012 and July 2014 (N = 208) in three secondary hospital-based pediatric practices in the Netherlands. The variation was considerable, with palivizumab being administered in 8%, 89% and 99% of patients of each practice. The main differences in counseling in the three practices concerned how the decision was presented (palivizumab is recommended vs. it is a preference-sensitive decision) and the way in which the benefits of immunization were described. In the practice with the lowest uptake the decision was presented as a personal choice based on the evaluation of benefits and risks and the benefits were described using the number needed to treat (NNT), specifying that 20 children need to be treated to prevent one hospitalization.

Van Beek and colleagues also assessed knowledge of healthcare providers about RSV infection, with a focus on children with Down's Syndrome [84]. Their study involved 53 participants, mainly pediatricians (79%), but also representative of patients' associations, nurses and caregivers. While most participants were aware that DS children have an increased risk of severe RSV infection, the guidelines available at the time of the study rarely included information about RSV prophylaxis in children with Down's Syndrome.

Finally, the last study on HCPs was conducted in Ireland in 2021 to examine nurses' knowledge of RSV and its prophylaxis and to explore their perceived potential barriers to palivizumab administration to children in the acute hospital setting [68]. Most of the 144 nurses participating were quite knowledgeable about RSV and its prevention, although many incorrectly believed that palivizumab is a vaccine and there were some gaps in knowledge about the eligibility criteria for it administration.

Since the effectiveness of palivizumab is short-termed, compliance with the monthly schedule is essential for ensuring immunization and six articles focused on this topic specifically. **Langkamp** and colleagues investigated parental factors associated with compliance in a sample of 385 parents of high-risk children eligible to receive palivizumab in the 1998-1999 season in the USA [57]. Twenty-two percent of eligible children missed one or more doses of palivizumab. Compliance was predicted mainly by parents' beliefs about its protective effects against RSV (compliant 67% vs. non-compliant 48%) and also interacted with the Medicaid status, with parents worrying the most and on Medicaid being more compliant. The main barrier to compliance was transportation issues (compliant 15% vs. non-compliant 35%). Similarly, **Robbins** and colleagues examined barriers that reduce adherence to RSV prevention in 143 parents in the USA during the 2001-2002 season [Robbins2002]. In this study infants receiving palivizumab were compared to those receiving RSV immune globuline (RSV-IG). The latter resulted in lower adherence (62% vs. 86%), higher infants' perceived distress and higher parents' distress than the former.

Anderson and colleagues examined the physicians' perspective about compliance with the monthly schedule in a sample of 100 pediatricians from multiple countries [33]. The most common barriers to compliance identified were caregiver inconvenience, distance to clinic, cost of prophylaxis, and lack of understanding of the severity of RSV. As interventions to improve compliance with palivizumab prophylaxis schedules participants recommended: provision of educational materials about RSV, reminders from hospital or clinic, and administration of prophylaxis at home to increase compliance.

Regarding the latter, **Ebersjö** and colleagues explored parents' perspectives about the possibility to administer palivizumab at home instead of in hospital or in an outpatient clinic in Sweden [41]. Fourty-three children eligible for palivizumab were recruited in a randomized pilot trial to evaluate safety aspects of at-home immunization. At the last visit, all parents of children who were immunized at home declared that they would prefer to receive the immunization at home if they could choose, whereas 70% of those who received it in hospital or clinic would prefer to receive it at home. The content analysis of their reasons to prefer home or hospital revealed three topics around the protection and monitoring of the infant, the health and well-being of the family, and the avoidance of suffering for the infant. The authors stress the importance of involving parents - who previously experienced neonatal intensive care - in the choice of place of immunization.

Erolu and colleagues analyzed the effectiveness of two nudge interventions in increasing compliance in children with chronic heart disease (CHD) in Turkey [42]. They recruited 229 infants and their caregivers in Turkey in the RSV season between 2020 and 2021. Participants randomized to the first intervention were called two days before their appointments and were asked to plan the appointment day. Indeed, implementing intentions has been shown to increase the likelihood that the planned behavior is carried out. Moreover, people tend to avoid changing the default option or deviating from a predetermined action plan. The second intervention provided biweekly information on the program's benefits as well as current adherence rate, leveraging the power of social norms and stressing the importance of. The other arm of the study was the control group. Relative to the control group (adherence 91%) the first intervention increased adherence (97%). When considering only children in their first RSV season (n=148), both interventions increased adherence (implementing intention 98%, social norms 97%) compared to the control group (88%).

Finally, **Pignotti** and colleagues examined factors associated to compliance with palivizumab in a sample of 216 parents of newborns in Italy between November 200 and April 2004 [73]. The results highlighted that overall compliance was 87%, with the main barriers identified being foreign-born or non-native speakers (compliance foreign parents 68%, native parents 89%). The authors recommend adequate information ensuring it is translated, although it is possible that also socio-economic factors are involved.

The remaining ten articles examined the perspectives of parents or parents to be on immunizing their children against RSV. One qualitative work conducted using semi-structured interviews explored the perspectives of 28 parents on administering nirsevimab to their healthy newborn in the USA by **Hinderstein** and colleagues [49]. Participants were recruited in the maternity and well newborn unit following delivery between November 2023 and February 2024, when nirsevimab was offered to all newborns, regardless of the vaccination status of the mother. Compared to other studies, RSV was more commonly known, but participants often showed gaps in knowledge about RSV prophylaxis, for instance by confusing it with vaccination and by considering it to be new and fear it was understudied. While recommendations from the pediatrician and worry about the disease fostered acceptance, some parents were concerned about side effects, wanted more time to decide, felt the maternal RSV vaccine was sufficient, and trusted their own prevention measures, resulting in deferral of nirsevimab.

Between February and June 2023, **Al-Jaid** and colleagues assessed parental knowledge of RSV infection and attitudes to infant immunization with monoclonal antibodies in a sample 606 parents in Saudi Arabia [31]. The majority of parents (83%) reported that their children had received all recommended vaccines included in the routine vaccination program and had a positive attitude toward vaccination in general (89%). While approximately half of parents had never heard about RSV and an additional 20% only knew a little about it, 55% reported that one of their children was diagnosed with RSV and/or bronchiolitis. The main reason to accept immunization was to protect infants, and 45% of parents would accept immunization as soon as possible, whereas the lack of knowledge about RSV and concerns for safety were the main barriers to immunization.

Two studies investigated attitudes and intentions to vaccinate against other diseases in addition to immunize against RSV [46,53]. One was conducted in the Netherlands and compared data collected in 2013 with data collected in 2022 on parents' acceptance of several vaccines, including a potential vaccine against RSV, among other possible expansion of the national immunization program [53]. The results showed that, while overall parents' attitudes towards vaccination were positive in both timeframes, there was a small but significant increase in parents indicating that their children were not fully vaccinated in 2022. Nonetheless, parents of children below 3.5 years held more positive attitudes towards potential future vaccines in 2022 than in 2013.

The other study was conducted in the USA at the end of September 2023, when immunization with nirsevimab started to be offered [46]. The sample expressing attitudes towards RSV immunization was

composed by parents with children under 8 months (n = 166), expectant mothers (n = 50) and fathers (n = 55), and respondents who indicated they were planning on getting pregnant over the next year (n = 345), whereas for COVID-19 and influenza the sample included all respondents who indicated they had children under age 18 (n = 1,538). Intention to vaccinate was highest against RSV (71%), followed by influenza (63%) and lowest for COVID-19 (40%). In line with the literature on vaccine hesitancy, for all positive predictors of intention to vaccinate were concerns about the disease, trust in health institutions, and previous vaccinations, whereas negative predictors were concerns about safety, necessity, and lack of information. Some predictors were specific for one or more diseases. For RSV intentions were higher for those who thought vaccines were important, but no association was found for risk perception, concerns about autism and gender of the responder.

Between February and June 2023, **Langer** and colleagues conducted their study in Germany, involving 191 parents of children up to 3 years and assessing their knowledge about RSV, perceptions of the disease, and attitudes towards general childhood vaccines and RSV immunization [56]. Most parents (84%) had never refused a routine vaccination for their children and 83% self-reported not to be hesitant about childhood vaccines in general, while 31% agreed that "it is better for children to receive fewer immunizations at a time". Only 3% of participants had never heard of RSV but 39% only heard about it. Sixty-eight percent would be willing to vaccinate their children against RSV, 24% were undecided, and 8% would refuse. Willingness to vaccinate against RSV was associated with general vaccine hesitancy and perceived severity of the disease. Parents whose children were born pre-term or had been hospitalized for an acute respiratory infection were more concerned about the disease.

Between March and May 2021, **Lee Mortensen** and Harrod-Lui examined parental knowledge about RSV and attitudes towards RSV immunization in a sample of over 5,600 parents of children below 2 years and expectant parents in several countries (China, France, Germany, Italy, Japan, Spain, UK, and US) [59]. Most participants declared that their children received all recommended childhood vaccines in their national routine immunization programs (74%) and intended to continue to do so (83%). Similarly to other studies, the awareness of RSV was rather low, with 36% of parents having never heard of it and 29% having heard the name but nothing else, whereas they were more aware of bronchiolitis, with only 8% of parents having never heard of it and 21% having heard the name but nothing else. After being informed about RSV and a potential new product to immunize against it, 60% of respondents would likely accept it if it was recommended as part of the immunization program and by the infant's HCP. In line with findings about vaccine hesitancy in general as well as those of other studies about RSV immunization, acceptance was most commonly promoted by parents' desire to protect their children, whereas potential side effects were the most common barrier to acceptance of the prophylaxis with antibody. When comparing answers from different countries, acceptance of the RSV immunization was lower among European and Japanese than among Chinese and Americans and HCP recommendation and inclusion in routine immunization programs was more relevant to their attitude. Moreover, parents with more than one child were more hesitant than new parents or parents to be. A focus on the French sample of this multi-countries study was reported in another article included in the review, stressing that the findings were supported by the actual uptake rate of nirsevimab (between 60 and 80%) following the introduction in September 2023 [58].

Sansone and colleagues recruited 404 parents of children parents in public kindergartens and nursery schools to examine their willingness to vaccinate their children with the RSV vaccine and the key predictors of this intention [79]. The study was conducted between April and November 2023 in Italy. While more than half (58%) of parents had heard about RSV infection, almost two-thirds (62%) would like to receive additional information about a vaccine against RSV for their child. A minority of participants had directly experienced in their past the diagnosis of bronchiolitis (12%) and hospitalization (8%). Nonetheless, probably due to the older age of the children at the time of survey (2.9 ± 1.3 years), participants showed low concern for RSV infection, with only 18% being very concerned about it, and concerns for the severity of the disease was not among the most frequently cited reasons to accept the vaccine (18%). Approximately half of participants (51%) would vaccinate their children; the most frequently mentioned reason to accept vaccination was to protect their children (67%) whereas the most common barrier was concern about possible side effects (60%).

Right during the start of the seasonal 2023–2024 immunization campaign against RSV, in September 2023 in Spain, **Zornoza Moreno** and colleagues recruited a sample of over 3 thousand parents of children below 2 years to examine their knowledge about RSV and bronchiolitis and attitudes towards nirsevimab immunization [90]. Similarly to the findings of other studies, the awareness of RSV was moderate (47%), whereas almost all participants (96%) were aware of bronchiolitis, with 38% declaring to have had previous

experience with it. The majority of parents were concerned about RSV severity (64%), its frequency in children (49%) and its contagiousness (48%). Despite this, only a minority of parents (11%) was aware of the new immunization. This might be due to the fact that their children were on average 1 year old and immunization was just starting to be offered when the study was conducted. Once informed, the vast majority of participants (97%) would accept nirsevimab immunization for their children. Similarly to the findings of other studies, the most frequently mentioned reason for accepting immunization was the protection of the infant when most vulnerable (78%) whereas the most frequently mentioned reason to refuse immunization was being concerned about safety and possible side effects (68%).

The acceptance of nirsevimab immunization during the first season of implementation in France was examined in almost 500 parents of newborns by **de Sentuary** and colleagues [40]. Among participants in the study, monoclonal antibodies were highly accepted (91.6%), confirming that the protection of the baby was the most common reason for accepting the prophylaxis, and that the lack of long-term data on nirsevimab was the most common reason for refusing it.

Finally, attitudes towards a possible future immunization against RSV were examined by **Wang** and colleagues in a sample of 2135 Chinese parents with children below 14 years old [87]. About one quarter of all participants had never heard of RSV and this proportion was higher among parents potentially involved in immunization (child < 1 year old). Nonetheless, 70.6 % of parents would vaccinate their child against RSV if a vaccine was available. Concern about vaccine's safety was the most common reason for refusing the RSV vaccine.
